# An adaptive design for updating the threshold value of a continuous biomarker

**DOI:** 10.1002/sim.7042

**Published:** 2016-07-14

**Authors:** Amy V. Spencer, Chris Harbron, Adrian Mander, James Wason, Ian Peers

**Affiliations:** a AstraZeneca, Global Medicines Development, Biometrics and Information Sciences, Mereside, Alderley Park, Macclesfield SK10 4TG, U.K.; b Roche Pharmaceuticals, 6 Falcon Way, Welwyn Garden City AL7 1TW, U.K.; c MRC Biostatistics Unit Hub for Trials Methodology Research, Cambridge Institute of Public Health, Cambridge Biomedical Campus, Forvie Site, Robinson Way, Cambridge CB2 0SR, U.K.

**Keywords:** adaptive design, biomarker-guided trial, threshold determination, Bayesian prediction, enrichment

## Abstract

Potential predictive biomarkers are often measured on a continuous scale, but in practice, a threshold value to divide the patient population into biomarker ‘positive’ and ‘negative’ is desirable. Early phase clinical trials are increasingly using biomarkers for patient selection, but at this stage, it is likely that little will be known about the relationship between the biomarker and the treatment outcome. We describe a single-arm trial design with adaptive enrichment, which can increase power to demonstrate efficacy within a patient subpopulation, the parameters of which are also estimated. Our design enables us to learn about the biomarker and optimally adjust the threshold during the study, using a combination of generalised linear modelling and Bayesian prediction. At the final analysis, a binomial exact test is carried out, allowing the hypothesis that ‘no population subset exists in which the novel treatment has a desirable response rate’ to be tested. Through extensive simulations, we are able to show increased power over fixed threshold methods in many situations without increasing the type-I error rate. We also show that estimates of the threshold, which defines the population subset, are unbiased and often more precise than those from fixed threshold studies. We provide an example of the method applied (retrospectively) to publically available data from a study of the use of tamoxifen after mastectomy by the German Breast Study Group, where progesterone receptor is the biomarker of interest.

## 1 Introduction

The development of drugs is rapidly changing from the traditional pipeline in which all patients with a broad phenotype are targeted by a single compound. It is now better understood that a broad phenotype is often a collection of similar phenotypes, and that biomarkers may be used as direct or indirect indicators of the subpopulation in which a particular drug is efficacious. For example, olaparib, a treatment which is effective in patients with BRCA-mutated breast cancer, does not appear to be effective in other patients with breast cancer [1–3]. There has been very little research into how biomarker ‘positive’ and ‘negative’ groups should be defined if the basic measure of a predictive biomarker is on a continuous scale. During clinical trials of targeted treatments, it is of interest to determine as early as possible whether there is a population/subpopulation in which the novel treatment is efficacious and how this target group should be defined. This might be aided by carrying out separate significance tests in different subpopulations and using these to inform the next phase [4,5] or by incorporating more efficient decision making through an adaptive design [6–8].

Some published methods of clinical trial data analysis incorporate the classification of a subset of patients benefitting from a treatment. For example, a Bayesian analysis which uses tree splitting based on factors including potential predictive dichotomous biomarkers [9] has been suggested. Algorithmic methods, including basic and cross-validated Adaptive Signature Designs [10,11], Virtual Twins [12] and SIDEScreen [13], also aim to choose the most appropriate subpopulation based on a set of biomarkers. With these methods, the biomarkers may be continuous and can be selected from a moderately large set of proposed factors.

There are also clinical trial designs which adaptively choose a subset during recruitment. The group sequential design for subgroups [14] could be used when the total population is made up of disjoint subsets. This may be appropriate when there is a single biomarker with a small number of levels or alternatively a small number of biomarkers which can each be classified as positive or negative, with the combined signature giving the subsets.

For a continuous biomarker, we define the true threshold or cut-off as the value which divides the population into two subsets, such that the treatment is considered to be effective in one of these subsets, and that this ‘positive’ subset is as large as possible. Ideally, these groups should be defined from clinical data, although frequently, the definition of these groups will be determined using separate data of questionable relevance, for example, from animal models. Even worse, the dividing threshold may simply be based on a value which is considered convenient. The biomarker-adaptive threshold design [15] is an algorithmic testing procedure which retrospectively aims to find the appropriate cut-point. This requires adjustments for multiple testing and could result in low power in an early phase clinical trial if a very small number of subjects are recruited with biomarker values above that cutpoint.

Tests of effect size in the ‘positive’ subset will be higher powered if this subset is enriched, and Simon and Simon [16] provide an adaptive enrichment solution. This study design assumes that there are two distinct response rates, one common to participants on the novel treatment with biomarker values below the true threshold and those on the control, and a higher one for those on the novel treatment with biomarker values above the threshold. An alternative adaptive design is suggested by Renfro *et al*. [17], which uses a set of models fitted across a reasonable range of cut-offs at the interim analysis. Depending on the results of these models, recruitment either continues from the whole population, from a subpopulation based on the best identified cut-off, or the trial is stopped for futility. The final tests for efficacy may also be based on either all recruited subjects or only those in the biomarker ‘positive’ subset, dependent on the stage two recruitment strategy.

We suggest an alternative adaptive recruitment design, the continuous biomarker-adaptive threshold trial (CBATT), which both selectively recruits from the start of the study and also updates the recruitment threshold to target a study population, which will have a statistically significant response rate. It has the advantage over the Renfro method [17] that all subjects will be used in the final test for efficacy, even if the recruitment strategy is changed at the interim. The basic study design contains a single arm, which is typical of early phase II oncology trials, although it could be modified to include a control arm. Recruitment is restricted using a preliminary threshold, and this recruitment threshold adapted following an interim analysis. Our adaptive recruitment design is ideal for an early phase clinical study as it aims to both demonstrate that there is a subpopulation in which the treatment is effective and estimate the most appropriate value of the biomarker to define the boundary of this subpopulation. This allows later phase studies to focus on this subpopulation alone.

In Section 2, we describe CBATT, stating a suitable interim analysis for a treatment with binary outcome and describing the eligibility criteria based on the value of the biomarker. We show a hypothesis test at the conclusion of the study and suggest a method for choosing an appropriate biomarker threshold to partition biomarker ‘positive’ and ‘negative’ patients in future applications of the treatment. In Section 3, we demonstrate the efficacy of our design in a number of scenarios, through a simulation study. In Section 4, we apply this design to data from the German Breast Study Group, investigating the use of hormonal therapy (tamoxifen) after mastectomy [18,19]. The simulations and analysis were carried out using R [20]. In Section 5, we discuss the potential of this design as well as its limitations and suggest ways in which this method could be modified.

## 2 An adaptive threshold trial design

In this section, we describe a single-arm trial design for a treatment with a binary outcome: either response or non-response. The objectives of the trial are to demonstrate the efficacy of the drug and to identify a subpopulation with a clinically relevant response rate. We assume a strong prior belief that a single biomarker measured on a continuous scale predicts the response rate in a monotonic relationship, with higher biomarker values relating to higher response rates. If the association is believed to be negative, the analyses should be adjusted appropriately.

**Figure 1:**
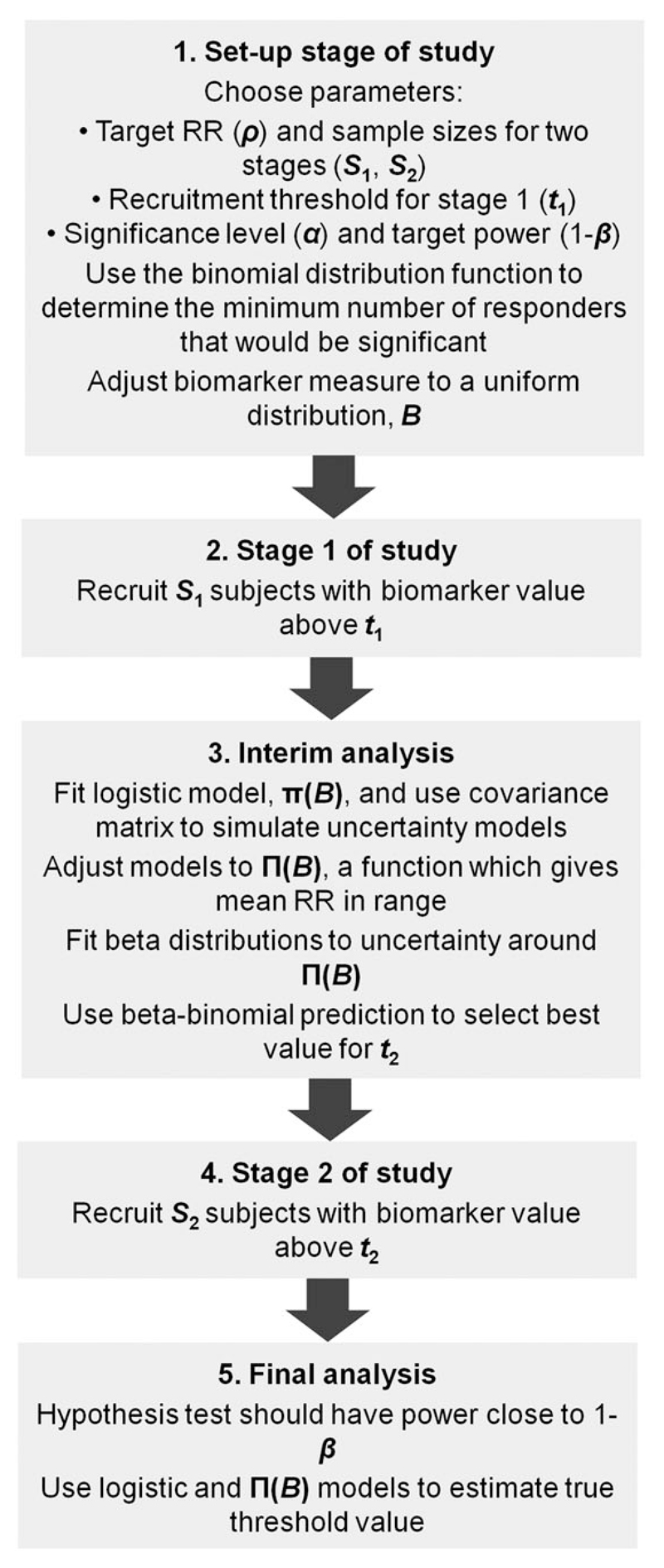
Flow diagram outlining the key steps in the continuous biomarker-adaptive threshold trial design, which are described in detail in Sections 2.1 to 2.3 of the main text.

Figure 1 shows a general overview of the study design, outlining the different stages and some of the key methods that are employed. The design requires the sample size, significance level and target power to be fixed at the beginning of the study. At the interim analysis, different possible recruitment thresholds are proposed for the second stage, the power that would be achieved if these were used is predicted and an updated recruitment threshold with a predicted power exceeding the target power chosen. If no predicted powers achieve the target power, there are several options, including early stopping for futility. Although we describe a two-stage study, this could be extended to include further interim analyses.

### 2.1 Overview of study design and notation

The primary aim of the study is to determine whether a subpopulation exists for which the response rate exceeds a pre-defined reference or ‘null’ response rate, *ρ*, justifying the progression of the drug in the clinical pipeline. The value of *ρ* should be chosen for clinical significance, for example, equal to or slightly higher than a historical response rate for a standard treatment. Part of the analysis involves integrating over the range of biomarker values (details in Supplementary material S1), and this is simplified by converting the measure of the biomarker to its estimated quantiles across the subject population, such that *B* ~ Unif[0,1]. The response rate at biomarker quantile *B* is written as *π*(*B*), and the response rate in the subpopulation with biomarker quantile ≥*B* is written as Π(*B*). Where T* denotes a possible biomarker quantile threshold value, the primary null and alternative hypotheses can be written

$$\begin{array}{l} H_{0}\;:\;\forall T^{*}\;:\;\Pi \left(T^{*}\right)\leq \rho ;\\ H_{\text{A}}\;:\;\exists T^{*}\;:\;\Pi \left(T^{*}\right)>\rho . \end{array}$$

We carry out a binomial exact test using all the subjects recruited to the trial. Due to our assumptions, this can be considered as a test at a single value of T*, and if the null hypothesis can be rejected at this value, then *H*_0_ can also be rejected. If this can be carried out, the secondary objective is to estimate the true threshold quantile of the biomarker, *T*, which defines the lower bound of the subpopulation of interest.

An adaptive design with a single interim analysis is used. The total sample size, *S*, and the stage specific sample sizes, *S*_i_ (*S* = *S*_1_ + *S*_2_), are fixed before the study begins. In order to increase the estimation accuracy of Π(*B*) at higher values of *B* using this fixed *S*, only subjects with *B*_j_ ≥ *t*_i_ are recruited, where *B*_j_ is the quantile value of the biomarker in subject *j* = 1,…,*S*, and *t*_i_ is the threshold in stage *i*. *t*_1_ should be chosen based on prior knowledge (refer to Section 3.4), and *t*_2_ should be chosen to aim to achieve a particular power in the hypothesis test at the end of the study, based on the results from stage 1. The significance level and target power in the hypothesis test are written as *α* and 1-*β*, respectively.

For each subject, an indicator of response, *x*_j_ = 0,1, is recorded. The overall and stage specific numbers of responses are denoted *X*_ob_ = Σ_j_ *x*_j_ and *X*_ob,i_ respectively. Because *S*, *ρ* and the significance level, *α*, are pre-specified, we can use the inverse of the binominal distribution function to calculate *X*_H_, the minimum number of responses that would produce a significant result, before the study begins. After stage 1, the remainder required is *X*_H,2_ = *X*_H_ − *X*_ob,1_. We also define *R*_H_ = *X*_H_ / *S* as the required response rate for the study to be significant and *T*_H_ the biomarker quantile value such that Π(*T*_H_ = *R*_H_).

### 2.2 Interim analysis

After choosing suitable values of the design parameters for the study and carrying out the stage 1 recruitment, an interim analysis with several steps is carried out.

As we assume that the response rate is monotonically increasing with increased levels of the biomarker, so that Π(max(*t*_1_,*t*_2_)) ≥Π(min(*t*_1_,*t*_2_)), we can use the binomial exact test *P*(*X*≥*X*_ob_ |*X* ~ Bi(*S*,*ρ*)) to test the following hypotheses at the end of the study:

$$\begin{array}{l} H_{0}^{*}\;:\;\Pi \left(\text{max}\left(t_{1},t_{2}\right)\right)\leq \rho ;\\ H_{\text{A}}^{*}\;:\;\Pi \left(\text{max}\left(t_{1},t_{2}\right)\right)>\rho . \end{array}$$

These hypotheses are not the same as *H*_0_ and *H*_A_, but if *H*_0_* is rejected, then *H*_0_ can also be rejected. At the interim, we use Bayesian beta-binomial prediction models to calculate the probability of observing the remaining number of required responses, *X*_H,2,_ in stage 2, based on what has been observed in stage 1. This probability is calculated at different values of T* and is the predicted power, 1 − *β*′_T*_, of a significant hypothesis test result if *t*_2_ = T*. The values of T* from which *t*_2_ will be chosen are given in the vector *t*_2_* and should be chosen by the clinical team such that they cover the full range of clinically meaningful values of the biomarker. It should also be considered that any value in *t*_2_* could be chosen as *t*_2_, so only recruitment thresholds that could be reasonably be used during stage 2 should be used (for example, *t*_2_ = 0.99 would result in only 1% of the disease population are being recruited, which may not be appropriate).

#### 2.2.1 Modelling response rate for population subsets based on stage 1 data

So long as the relationship between *B* and the probability of response can be assumed to be smooth and monotonic, it is reasonable to model it using a logistic regression model, π(*B*) = exp(*z*) / (1 + exp(*z*)), with linear predictor *z* = *δ*_0_ + *δ*_1_*B*. A heavier tailed curve may be better modelled by a probit model, for example, but this design is not powered to choose between two types of model. Fitting the data from stage 1 to such a model gives unbiased estimates of the response rates at fixed values of *B*, including *B* < *t*_1_. However, Π(*B*), the response rate in a population subset is more meaningful than π(*B*) in this trial design, and we show in Supplementary material S1 how this function can be derived from the logistic model:

$$\Pi \left(B\right)=\text{ln}\left(\frac{1+\text{exp}\left(\delta_{0}+\delta_{1}\right)}{1+\text{exp}\left(\delta_{0}+\delta_{1}B\right)}\right)/\delta_{1}\left(1-B\right).$$

To account for the uncertainty in the maximum-likelihood estimates of the fitted model coefficients, (*δ̂*_0_, *δ̂*_1_), and therefore in *π̂*(*B* and Π̂ (*B*), we generate 1000 realisations of hypothetical model coefficients using the Fisher information matrix from the fitted model:

$$\left(\begin{array}{l} {\tilde{\delta }}_{0}\\ {\tilde{\delta }}_{1} \end{array}\right)˜MVN\left(\left(\begin{array}{l} {\hat{\delta }}_{0}\\ {\hat{\delta }}_{1} \end{array}\right),\left(\begin{array}{cc} \sigma_{0}^{2} & \sigma_{01}\\ \sigma_{01} & \sigma_{1}^{2} \end{array}\right)\right).$$

Each realisation of $\left({\tilde{\delta }}_{0},{\tilde{\delta }}_{1}\right)$ gives an estimate of the biomarker subgroup model, $\tilde{\Pi }$(*B*), given *B* ~ Unif[0,1].

#### 2.2.2 Beta-binomial prediction

For each of the *k* potential threshold values in *t*_2_*, we can generate 1000 $\tilde{\Pi }\left(B=t_{2,\text{k}}^{*}\right)$ values using the different $\left({\tilde{\delta }}_{0},{\tilde{\delta }}_{1}\right),$ which are estimates of $\Pi \left(t_{2,\text{k}}^{*}\right)$ and take into account the uncertainty from the stage 1 data. To these values, we fit a beta distribution $\tilde{\Pi }\left(t_{2,k}^{*}\right)˜B\left(a_{t_{2,k}^{*}},b_{t_{2,k}^{*}}\right)$ and predict the number of responses in stage 2 using $X_{t_{2,k}^{*}}˜\mathrm{BBi}\left(S_{2},a_{t_{2,k}^{*}},b_{t_{2,k}^{*}}\right).$ We use these beta-binomial distributions to calculate the predicted power, $1-{\beta^{\prime }}_{{\text{t}}_{2,\text{k}}^{*}}=P\left(X_{{\text{t}}_{2,\text{k}}^{*}}\geq X_{\text{H},2}\right).$ The recruitment threshold for stage 2, *t*_2_, is the minimum $t_{2,\text{k}}^{*}$ such that $1-{\beta^{\prime }}_{{\text{t}}_{2,\text{k}}^{*}}\geq 1-\beta .$

Use of this beta distribution is convenient because it is a conjugate prior, is empirically based on the stage 1 data and can easily accommodate different distributional assumptions of location and dispersion. The latter is particularly appropriate when we want to update our probabilities. We investigated whether the use of beta distributions is suitable by comparing it with using densities derived from Markov chain Monte Carlo (MCMC) methods with various simulated datasets. We found that the beta density was usually close to the MCMC density, and the two methods tended to give similar final results. An example illustration is given in Supplementary Figure 1. The MCMC method is more computationally intensive but allows our method to be generalised where a logistic model and beta densities cannot be assumed.

It may be the case that none of the values in $t_{2,}^{*}$ have the property $1-{\beta^{\prime }}_{{\text{t}}_{2,\text{k}}^{*}}\geq 1-\beta ,$ that is, no threshold achieves the desired predicted power. This could be used as a strict stopping rule. When the largest $t_{2,\text{k}}^{*},t_{2,\text{max}}^{*},$ has an acceptably high $1-{\beta^{\prime }}_{{\text{t}}_{2,\text{max}}^{*}},$ it may be decided to progress with $t_{2}=t_{2,\text{k}}^{*}.$ Guidelines for the application of such rules are given in Section 2.4.

### 2.3 Analyses after stage two

#### 2.3.1 Significance test for demonstrating efficacy

If the trial is not stopped at the interim, *S*_2_ subjects with *B*_j_≥*t*_2_ will be recruited and observed to see how many respond to the treatment. The binomial exact test *P*(*X*≥*X*_ob_|*X*~ Bi(*S*,*ρ*)) should produce a significant result at level *α* with a probability of approximately 1 − *β*′_t_2__, allowing for $H_{0}^{*}$, and therefore also *H*_0_, to be rejected. This result supports proceeding to the next stage of clinical development, but we also wish to be able to define the subpopulation in which the drug should be tested.

#### 2.3.2 Estimating the true threshold

The full dataset can be modelled in the same way that the stage 1 data were modelled at the interim analysis: fitting a logistic regression model to *π*(*B*), generating 1000 realisations of the regression coefficients of $\left({\tilde{\delta }}_{0},{\tilde{\delta }}_{1}\right)$ and using these to calculate 1000 $\tilde{\Pi }$ (*B*) estimates at each T*. If Π̂(*B*) is calculated in the same way using (*δ̂*_0_, *δ̂*_1_), the maximum-likelihood estimate of the true threshold, *T̂*, is the value of *B* at which min(|Π̂(*B*) − *ρ*|) occurs. The distribution of min(|$\tilde{\Pi }$(*B*) − *ρ*|) can be used to find values of *B*, which give confidence intervals around *T̂*. In Section 3.3, we show that *T̂* is an unbiased estimate of *T*.

### 2.4 Implementing either a fixed or adaptive threshold design

The adaptive design is based around a target predictive power (1-*β*), and as discussed in Section 2.2.2, there are several options for rules if there is no $t_{2,\text{k}}^{*}$ such that $1-{\beta^{\prime }}_{{\text{t}}_{2,\text{k}}^{*}}\geq 1-\beta .$ Here, we suggest three versions of the adaptive design, one with a strict stopping rule at the interim, one with no stopping rule and one which is a compromise between these. We also suggest two fixed threshold designs, one with a stopping rule and one without, which we use to compare to the adaptive methods in the simulations in Section 3. Many studies include futility stopping rules at an interim analysis. An example of a biomarker-based design which does this is SWOG S0819 [21], which includes rules to stop early for futility both in the EGFR FISH+ and non-positive groups. The paper by Redman *et al*. includes some discussion around the inclusion of futility monitoring for the subgroups defined by the biomarker.

Adaptive design 1 (AD1) is the adaptive design which uses, in stage 2, the lowest threshold with a predicted power greater than 1-*β*. If no such threshold exists, the study is stopped early at the interim. Adaptive design 2 (AD2) is a variation on AD1. In AD2, if there is no threshold with predicted power greater than 1-*β*, the maximum threshold $\left(t_{2,\text{max}}^{*}\right)$ will be used in stage 2, but only if the predicted power at this threshold is greater than another predefined value, *γ*. Otherwise, the study will be terminated. AD1 is a special case of AD2 with *γ* = 1-*β*. Another special case is adaptive design 3 (AD3), where *γ* = 0, meaning, it has no minimum predicted power limit to using $t_{2,\text{max}}^{*},$ so no trials stop early.

Fixed design 1 (FD1) uses a fixed threshold throughout the study. However, an interim analysis is carried out, which does not use the biomarker data, and the following stopping rule is used after stage 1: stop if P(*X*_ob_≥*X*_H_|*X*_ob,2_ ~ BB_i_(*S*_2_, *X*_ob,1,_ *S*_1_ − *X*_ob,1_)) < 1-*β*_FD_. Fixed design 2 (FD2) does not stop trials early.

## 3 Simulation study

To test our study design, we simulated data from a number of possible scenarios. Our full R [20] simulation code is provided in the Supplementary material Section S2. We compared the simulated study outcomes that would result from using the designs described in the preceding texts.

### 3.1 Simulated data and analysis parameters

Data were simulated from a population in which the true underlying response rate can be described by a logistic curve, with linear predictor *z* = *δ*_0_ + *δ*_1_*B*. We assumed that the biomarker distribution was well characterised and can be represented by the quantile values, so that *B* ~ U[0,1], and chose values of *δ*_0_ and *δ*_1_ (the log odds ratio), which resulted in particular values of *T* or *T*_H_, the true threshold as described in the preceding texts relating to the response rate *ρ* or *R*_H_ respectively. For the various simulation scenarios, we varied: values for *S*_1_ and *S*_2_, the sample sizes in stages 1 and 2 and *t*_1_, the lower recruitment threshold in stage 1. Other parameters were kept constant, with *α* = 0.05, 1-*β* = 0.8, and where there was a true threshold, *ρ* = 0.4. 5000 iterations were run using each particular set of values.

For each iteration, data for stage 1 were generated using *B*_i_ ~ *U*[*t*_1_, 1]; *X*_i_ ~ Bern(π(*B*_i_)). We allowed 20 possible values that *t*_2_ could take, $t_{2}^{*}=\left(0,0.05,0.1,\;\ldots \;,0.95\right).$ The predicted power was calculated for each of these values. Data were then generated for 20 possible stage 2 s, each using a different value of *t*_2_. This allowed us, post-simulation, to consider which 5000 of the 20 * 5000 potential stage 2 s would have occurred under different interim decisions, and how these choices affect power.

The main focus is the comparison of AD1 with 1-*β* = 0.8 with FD1 with 1-*β*_FD_ = 0.2. The fixed threshold in FD1 is equivalent to *t*_1_ in AD1, so the stopping rules are in AD1, stop if *p*(success) < 1 − *β* = 0.8 ∀ *t*_2,k,_ and in FD1, stop if *p*(success)<1 − *β*_FD_ = 0.2 | *t*_2_ = *t*_1_. However, these probabilities of success are not calculated in the same way, with the FD1 method using the observed values directly rather than the output of logistic modelling and simulations.

### 3.2 Results of a basic adaptive study for simulation scenarios

#### 3.2.1 Type-I error rate

The type-I error rate (TOER) can be examined by looking at the proportion of significant study results in scenarios where *H*_0_ is true. Using *S*_1_ = *S*_2_ = 50 and *t*_1_ = 0.5, we analysed simulated data from a number of underlying logistic curves with Π(0.95) just under *ρ* = 0.4, and the results of these simulations are give in Table I. The overall TOER is very low for both designs in all of these scenarios and is usually slightly smaller when the adaptive design is employed. Although the TOER in the completed studies is often higher when using the adaptive design, it should be taken into account that this is a proportion of a much smaller number of the 5000 simulated studies reaching completion. For example, with Π(0) = 0.39, 91 of 454 completed studies had significant results when using the adaptive design compared with 143 of 1095 using the fixed design. This highlights that the results are affected by the different stopping rules applied in the two designs (refer to Sections 2.4 and 3.1).

The TOER is not specifically controlled at the chosen *α* value (0.05), but importantly, it is bounded at this level and is conservative. This is because the number of responders needed to get a significant response (*X*_H_) is always 49, but the probability of observing this number increases with Π(0). Also, prediction at the interim analysis causes the majority of studies to stop early, some of which would have produced significant results if they were to continue. These low TOERs suggest that higher values of *α* could be implemented, whilst maintaining overall control of TOER, thus increasing the power of the method.

The last two columns of Table I contain the ratio of participants that would have to be screened (assuming a representative distribution of the biomarker in the sample) in the adaptive design studies compared with the fixed designs in order to recruit the required sample size. By ‘screening’, we mean measuring the biomarker value in otherwise suitable study participants to find those with *B* > *t*_i_. Using a fixed design with *S*_1_ = *S*_2_ = 50, *t*_1_ = *t*_2_ = 0.5, the approximate number of subjects screened would be 100 for a study stopped at the interim and 200 for one which reaches completion. Taking into account the completed studies only, the given ratio is >1 when the simulated adaptive studies usually have *t*_2_ > 0.5 and increases with the average value of *t*_2_.

#### 3.2.2 Non-null scenarios with a range of true thresholds

We next considered how the results of AD1 and FD1 with a single set of parameters might vary under different scenarios where there is a biomarker effect and *H*_A_ is true but kept most of the parameters the same as those in the null scenarios in the preceding texts. We considered three magnitudes of the biomarker effect: *δ*_1_ = 3, *δ*_1_ = 6 and *δ*_1_ = 9, and we simulated from curves with these *δ*_1_ values, which also had particular true threshold (*T*) values or hypothesis-testing thresholds (*T*_H_). In Table II, we summarise the curves using Π(*B*) at *B* = 0 and 0.95. To demonstrate the range of simulated scenarios investigated, we show the logistic curves and corresponding Π(*B*) functions for those in Table II in Supplementary Figure 2. The outcomes are two measures of power, the stopping rate and a ratio of subjects screened. The overall power is the proportion of the total 5000 iterations that resulted in a signficant outcome in the hypothesis test. The power for the completed studies is the proportion of studies not stopped at the interim, which went on to give a significant result. The proportion of studies stopped early (stopping rate) increases with the true threshold, as the number of responses in stage 1, *X*_ob,1_, will generally decrease. Also, the proportion of studies stopped early at a particular value of *T* varies more (dependent on *δ*_1_) in AD1 than in FD1.

Both the overall power and the power for the completed studies are greater when using AD1 rather than FD1 for all simulations with *T* > 0.3. The power of the adaptive design is generally closer to 0.8 than that of the fixed design. Where *T* << *t*_1_, using *t*_2_ = *t*_1_ = 0.5 (as in the fixed design) is likely to result in a power >0.8, so the adaptive design will usually choose *t*_2_ < *t*_1_. In fact, for the simulations with *T* = 0.2 and with *δ*_1_ = 3, 6 and 9, the median values of *t*_2_ in the adaptive design are 0.5, 0.4 and 0.3 respectively. This allows the stage 2 data to generate a more informative biomarker response curve.

For a particular *δ*_1_, the powers given in the table generally increase as *T* decreases. For FD1, this is expected, because a lower *T* means that recruiting subjects with *B* > *t*_1_ = 0.5 will result in an overall higher response rate. For AD1, the interim rule is to only continue the study using *t*_2_ if the predicted power for this *t*_2_ is ≥0.8. This would suggest that the power in the completed studies should consistently be slightly higher than 0.8. We observe cases where the power is much higher than 0.8, and this occurs when *T* << *t*_1_, as in this situation, sometimes even the smallest $t_{2,\text{k}}^{*}$ has predicted power >> 0.8. However, we also see that when *T* is high and/or *δ*_1_ is low, the power in the completed studies can be much lower than 0.8. This can be explained in terms of asymmetric decisions being made between underestimates and overestimates of power for different $t_{2,\text{k}}^{*}$ and is discussed further in Section 5.2.

#### 3.2.3 Scenarios in which the biomarker is not predictive

It may be the case that the biomarker is not predictive of treatment response, and subjects with all values of *B* have the same probability of response (Π). Table III contains the results for scenarios in which some subjects respond, but there is no biomarker effect. In some cases Π > *ρ*, so *H*_A_ is true, but *δ*_1_ = 0 and *T* = 0. The approximate number of subjects that would need to be screened in any of these cases is fairly similar no matter which of the two designs is used. However, as the response rate increases, the proportion of studies producing significant results (overall and amongst completed studies) increases for both study designs. The proportion of significant studies is very low when *H*_0_ is true (Π < *ρ*) and very high when Π > > *ρ*. In fact, when Π = 0.35, almost all studies are stopped at the interim (0.934 for the fixed design and 0.975 for the adaptive design), whereas when Π = 0.65, very few are stopped (0.001 and 0.018 respectively). Only in the cases where Π > *R*_H_ = 0.49 do we see significant proportions in the completed studies of 0.8 or higher.

For this subset of scenarios, it is possible to calculate the stopping rate and the probability of significance exactly. In Supplementary material S3, we demonstrate how these compare closely to the results from the simulation.

### 3.3 Estimating T

The secondary aim of the adaptive study design is to estimate *T*, which can be carried out even if the result of the hypothesis test is non-significant. In each simulation, we found the bias in the estimate using bias = *T̂* − *T* and summarised the uncertainty in *T̂* using confidence intervals (CIs) based on the 1000 $\tilde{\Pi }$(T*) estimates in that study. We consider the median bias in each set of 5000 simulation iterations, as well as the interquartile range of the estimates. For studies that did not reach completion, we used the estimates from the logistic regression fitted at the interim analysis.

**Figure 2:**
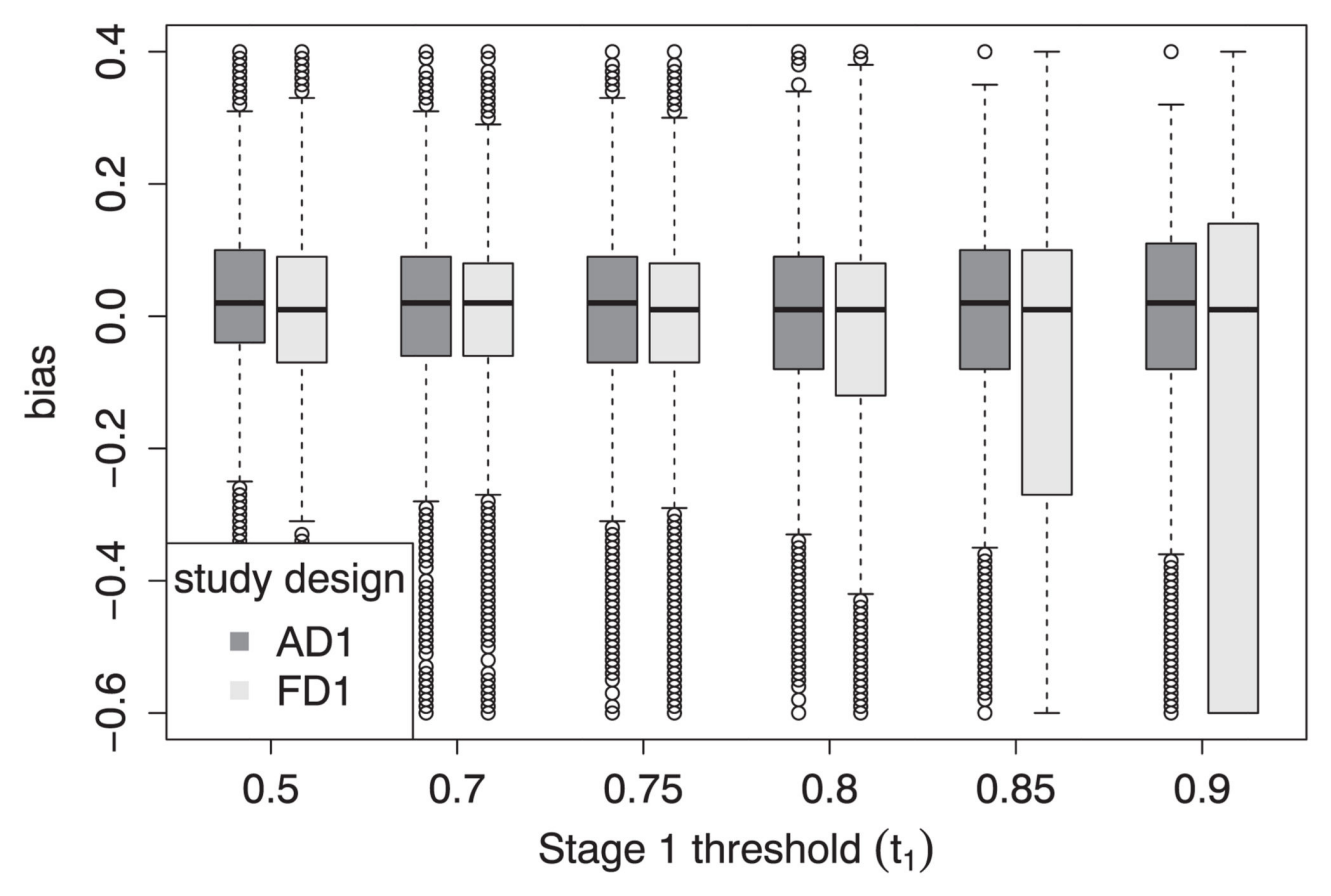
The bias in 5000 iterations of a simulated study dependent on the stage 1 threshold (*t*_1_) and the study design where AD1 is adaptive design 1 and FD1 is fixed design 1. The data are generated from a logistic model with *T* = 0.6 and *δ*_1_ = 6. Other parameters are fixed at *α* = 0.05, 1-*β* = 0.8, *ρ* = 0.4 and *S*_1_ = *S*_2_ = 50.

Across all simulations, the average bias was very close to 0. For example, for the scenarios shown in Table II, the median bias in *T̂* from the adaptive design analyses was always in the range (−0.01, 0.03), which is very similar to the equivalent range from using the fixed design of (−0.01, 0.02). The interquartile range of *T̂* generally increases slightly as the value of *t*_1_ increases, as less of the biomarker’s dynamic range was studied in stage 1, but in most cases, this distribution was similar no matter whether AD1 or FD1 was used. The exception to this is when *t*_1_ (the stage 1 threshold in AD1 or fixed threshold in FD1) is very close to 1, particularly if *T* is not. This can be observed by simulating a single scenario in which *T* = 0.6, *δ*_1_ = 6, *S*_1_ = *S*_2_ = 50, *ρ* = 0.4, *α* = 0.05 and a range of different values for *t*_1_. Boxplots of the bias in *T̂* observed in these simulations are given in Figure 2. These show that for larger initial thresholds, the interquartile range for *T̂* was generally smaller in AD1 than in FD1, for example, 0.17 compared with 0.20 when using *t*_1_ = 0.8 and 0.19 compared with 0.74 when using *t*_1_ = 0.9. Only a small proportion of studies were stopped in these simulations, so these estimates mostly rely on completed studies; the main difference being that in AD1, the majority of studies had *t*_2_ < *t*_1_. Fixing *t* at a very high value (as in FD1) means that the estimates based on an extrapolated fitted model will often be very poor. The estimates are improved if some data come from lower values, for example, where *t*_1_ = 0.9, the median *t*_2_ in AD1 was 0.75 (other quartiles: 0.50, 0.85). The uncertainty in the individual study estimates of *T̂* is similarly affected in these examples. The median width of the 50% CI in the simulations with *t*_1_ = 0.8 is 0.18 using the fixed design, 0.13 using the adaptive design and 0.33 and 0.14 respectively when *t*_1_ = 0.9.

### 3.4 *Choice of S* and *t*_1_

For deployment of the method in any particular scenario, we recommend the use of simulations to understand the power and operating characteristics of any proposed design and sample size. In order to aid practitioners, we have included our R [20] simulation code in Supplementary material S2 for this purpose.

Power increases with *t*_1_ (Supplementary Figure 3b), but so does the uncertainty in *T̂*, meaning that there is a trade-off when choosing this value. Time restrictions on the study also introduce a trade-off between *S* and *t*_1_, as recruitment will not be from the whole population, and accrual rates in each stage depend on *t*_i_. If *T*_H_ can be estimated with some confidence prior to the study, then we would suggest a value close to this for *t*_1_, if it provides an acceptable screening rate. Supplementary material S4 examines the effect of changing the timing of the interim analysis. It appears that in general, using *S*_2_ = *S*_1_ will at least give close to the highest possible power or the desired power (1-*β*) in the case *t*_1_ > > *T*, although if a low value of *t*_1_ is being used for some reason, it may be advantageous to place the interim early in the study.

### 3.5 Using alternative study designs

In Section 2.4, we describe some alternative study designs implementing the same basic methods. These alternatives might allow more studies to reach completion, and in particular, more studies in populations where a subpopulation of interest does in fact exist (increasing the true positive rate). To investigate the extent of this effect, we also applied adaptive AD2 and AD3 and FD2 to the simulated datasets. With the adaptive designs, we continued to use 1-*β* = 0.8, and with AD2, we set the minimum predicted power with which a trial would be allowed to continue, *γ*, at 0.5.

**Figure 3:**
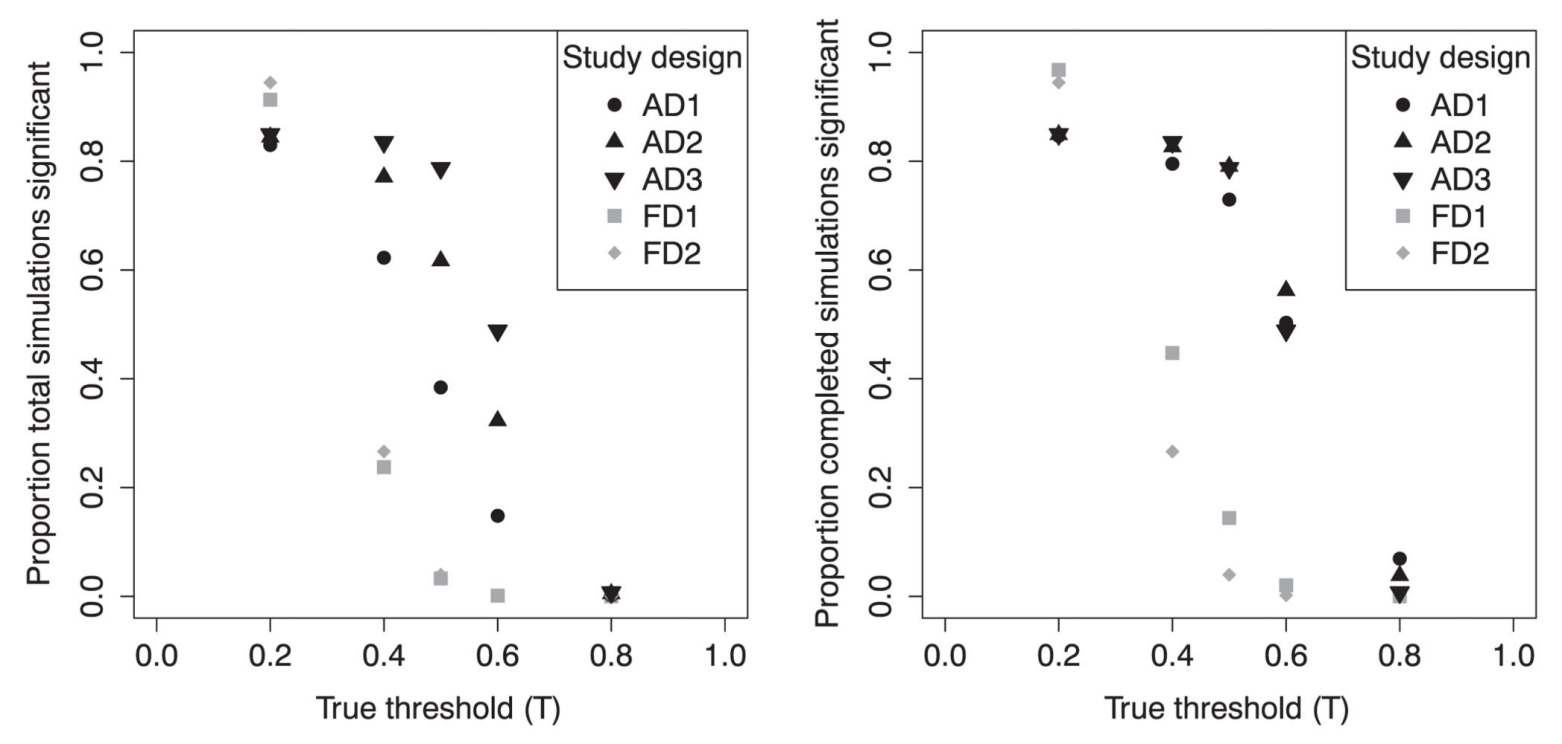
The power of different study designs using *t*_1_ = 0.5, *α* = 0.05 and 1-*β* = 0.8 or 1-*β*_FD_ = 0.2, *ρ* = 0.4 and *S*_1_ = *S*_2_ = 50, with *δ*_1_ = 6, but a variety of true thresholds. We give (a) the overall power (proportion of 5000 simulation iterations which have significant hypothesis test results) and (b) the power within completed studies (those iterations which are not stopped at the interim).

Figure 3 displays the observed powers for 5000 iterations of five simulated scenarios, each analysed using the five different study designs. In Figure 3a, we see the overall powers, and in Figure 3b, we see the powers within the completed studies. Notice that the same results are given for AD3 and FD2 in both figures, as these designs do not have stopping rules. As we previously observed for AD1 and FD1, all of the adaptive designs generally have power closer to 0.8 than the fixed designs. Allowing more studies to reach completion increases the overall number that produce significant results, so we see that in Figure 3a, power(AD3) > power(AD2) > power(AD1) for all values of *T*. It is somewhat surprising, though, that this is also the case at the lower values of *T* when we adjust for stopping at the interim. We would expect the designs which stop fewer studies to have, on average, lower predicted powers and therefore also lower observed powers. This is explained by the imbalance between over-prediction and under-prediction of power (refer to Section 5.2), which will have the largest effect in AD1 (where predicted powers need to be highest for the study to continue), and the fact that at these lower values of *T*, the target predicted power of 0.8 is likely to have only just been missed in a large proportion of studies (in some cases due to under-prediction).

The results in Figure 3 might at first seem to argue against using strict stopping rules, but such rules allow early detection and stopping for futility when the null hypothesis is true. Take, for example, simulations of three scenarios with *H*_0_ true (Π(0) = 0.36, Π(0.95) just below 0.4) and using *t*_1_ = 0.5, 0.7 and 0.9; the rest of the parameters are the same as those simulations in Figure 3. All three designs consistently resulted in overall type-I error rates <0.04 and completed study TOERs <0.12, but whereas AD3 would automatically take all studies to completion, AD2 stopped 77–84% of the studies, and AD1 stopped 84–95% of the studies at the interim. In practice, these design parameters can be adjusted to achieve an appropriate trade-off between false-positive and false-negative risks for the scenario under which the study is being performed.

## 4 Case study

### 4.1 Data

To illustrate our method, we applied it retrospectively to the data from the German Breast Study Group [18,19]. This dataset contains recurrence free survival (RFS) information on 686 patients with breast cancer who had undergone a mastectomy, but we focus only on those given tamoxifen, a hormonal treatment (*n* = 246). The dataset includes baseline progesterone receptor (PR) measurement for all subjects. In the original analysis, the continuous values were not used,but were instead split into positive or negative, with positive classed as PR ≥20 fmol/mg. We wished to investigate whether there is a subpopulation with 65% RFS at 1500 days after mastectomy which can be predicted by PR. For this retrospective analysis, we therefore define a responder as a subject who is recurrence free after 1500 days and a non-responder as a subject for whom recurrence or death occurred within 1500 days. We realise that such a long-term outcome is not typical of an adaptive design study where an earlier endpoint is required, but this dataset is used to provide an illustration of the principle of the method. A number of subjects were lost to follow-up within 1500 days, so their data had to be discarded, leaving a total of 176 subjects in the tamoxifen group.

We used the full set of 686 PR values to estimate the biomarker distribution across the patient population and transform these to *B* ~ Unif[0,1]. As well as *ρ* = 0.65, we set the initial study parameters at *S*_1_ = *S*_2_ = 35, *t*_1_ = 0.35 (which is equivalent to subjects with PR ≥ 15 fmol/mg), *α* = 0.05, 1-*β* = 0.8. In order to imitate the conditions of an ongoing study as closely as possible, we ‘recruited’ subjects by ascending ID numbers. Our ‘adaptive design study’ therefore included the first 35 subjects with PR ≥15 fmol/mg and the subsequent 35 subjects based on *t*_2_, which was determined at the interim analysis.

### 4.2 Results

*X*_H_ = 53/70 responders (subjects who are recurrence free at 1500 days) needed to be observed in order to achieve a significant test result, with the alternative hypothesis RR >0.65. In stage 1, we observed *X*_ob,1_ = 26/35 responders, meaning that in stage 2, the target was *X*_H,2_ = 27. We fitted a logistic regression model and used the parameter estimates and covariance matrix to fit beta distributions around Π̂(T*), where $t_{2}^{*}=\left(0,0.05,0.1,\ldots ,0.95\right).$ Using the beta-binomial predictions at each of these potential thresholds, the minimum $t_{2,\text{k}}^{*}$ with predicted P(*X*_ob,2_ ≥ 27) ≥ 0.8 was 0.55.

After completing the ‘recruitment’ using *t*_2_ = 0.55 (equivalent to PR ≥ 47 fmol/mg), there were exactly 27 further responders, meaning that the binomial exact test on the 70 selected subjects indicated that there is a subgroup with 65% RFS in the tamoxifen-taking population (*p* = 0.037). This subgroup is defined by higher progesterone receptor counts, and using the Π(*B*) function, we can estimate that the relevant threshold in terms of *B*, the progesterone receptor quantiles, is 0.2 (75% CI = (0, 0.35)). This is equivalent to 4 fmol/mg (75% CI = (0, 15)).

**Figure 4:**
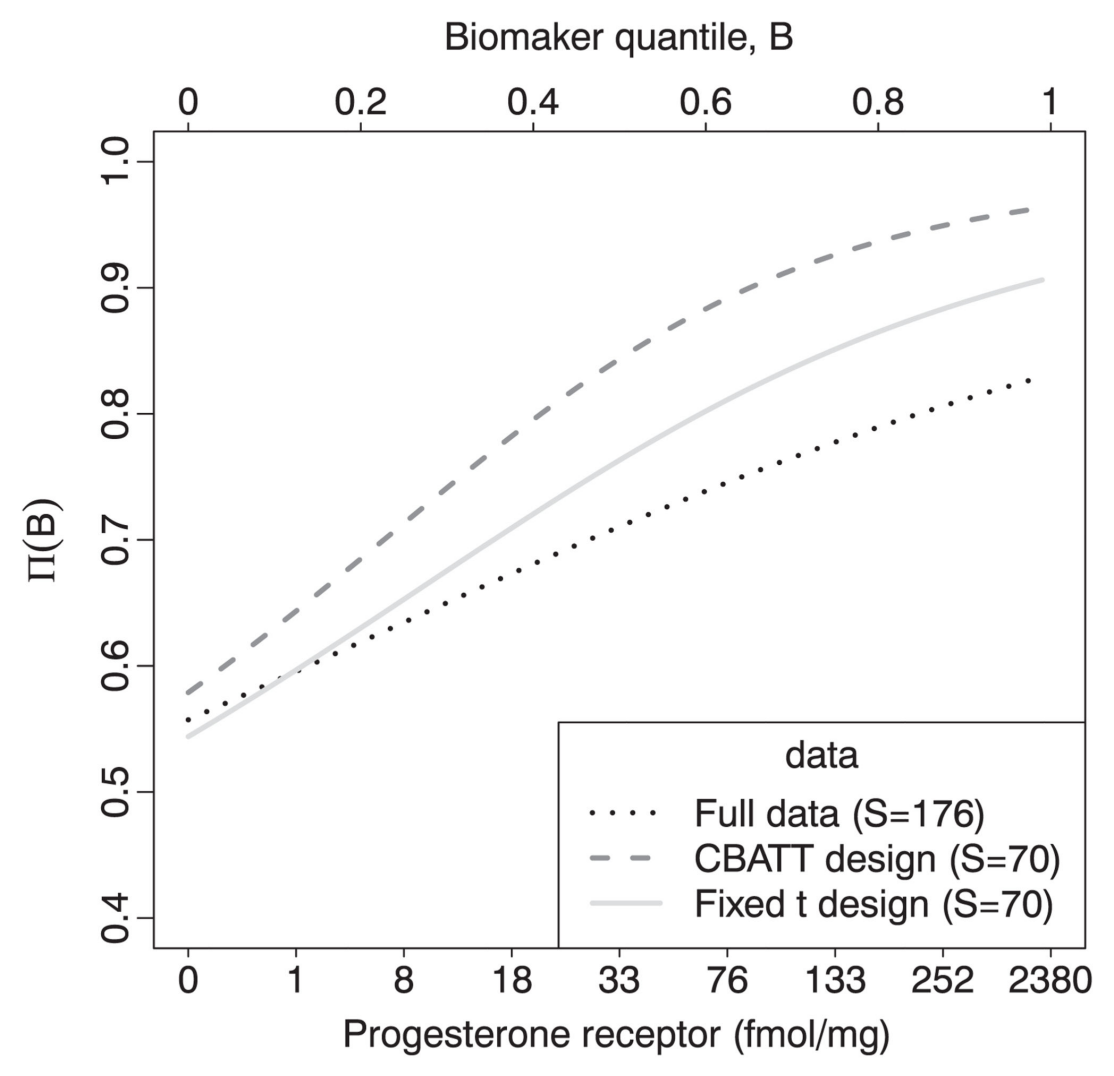
Estimated average recurrence free survival rate at 1500 days after mastectomy for the subpopulation, with progesterone receptor levels above those given on the x-axis. This was calculated with the full available data (*S* = 176) as well as with two ‘retrospective recruitment’ studies with *S* = 70, *ρ* = 0.65, *α* = 0.05, 1-*β* = 0.8 and *t*_1_ or *t* = 0.35.

Table IV compares the results of this retrospective CBATT study to those of an alternative retrospective subgroup analysis where the first *S* = 70 subjects with *B* > *t* = 0.35 are ‘recruited’ (=*t*_1_ in the adaptive design). In this case, a fixed recruitment threshold of 0.35 resulted in 48 responders (*p* = 0.312). The best estimate of the true threshold value we are able to obtain comes from analysing the data of all subjects on hormonal therapy whose survival data were not censored before 1500 days. The point estimate is 11 fmol/mg, and this is within the 75% CIs of the estimates from both of the smaller subsets. The fitted Π(*B*) curves from both retrospective analyses and the full data analysis are shown in Figure 4.

## 5 Discussion

### 5.1 CBATT design and implementation

We have described an adaptive study design for single-armed clinical trials in which there is a potentially predictive biomarker measured on a continuous scale which does not have a pre-defined cut-off for ‘biomarker positive’ subjects. Defining the ‘true threshold’ as the biomarker value in the preceding texts in which the subjects have the desired response rate, our design recruits subjects from a subset of the population using an initial biomarker threshold. This recruitment threshold is adapted at an interim analysis in order to optimise the information obtained about the biomarker response relationship whilst also ensuring the desired level of power in the study. The interim analysis has been developed using Bayesian beta-binomial prediction models, which predict that the power of binomial exact tests for response rate at the study conclusion using different thresholds that may be used in stage 2.

The primary focus of the design is around testing for efficacy in a subpopulation defined by biomarker values. In order to justify further development of a drug in more expensive later phase trials, robust demonstration of a drug’s efficacy is required. Once the efficacy of the drug has been established, detailed understanding of factors, such as the patient population in which that drug is most effective will increase the probability of successful development. Our method also allows for unbiased estimation of the biomarker-response curve, and we would advocate the presentation of such results alongside hypothesis test results.

For ease of presentation, the method we have demonstrated uses a mixture of Bayesian and frequentist methodologies. However, it would be relatively simple to work in a completely Bayesian framework, using Bayesian logistic regression modelling and Bayesian hypothesis testing approaches.

Over a range of simulations with different true thresholds, the adaptive design with a stopping rule (AD1) consistently outperformed a fixed design with a stopping rule (FD1). We also demonstrated other versions of the adaptive design (AD2 and AD3), which provide higher power still at the cost of an increased probability of proceeding to study conclusion when the null hypothesis is true. Generally, in simulations, the adaptive designs resulted in a power closer to the predicted power than fixed designs both with (FD1) and without (FD2) a stopping rule when the true threshold >0. These designs also produced similar power in simulated scenarios for which the reference response rate was observed overall, but the biomarker was not predictive, and they had comparable type-I error rates. At the end of the study, the true threshold can be estimated, and using an adaptive design results in an unbiased estimate. In situations when the initial recruitment threshold is greater than the true threshold, it also gives less uncertainty in the final threshold estimate than a fixed design.

For the method to be effective, the approximate distribution of the biomarker in the subject population must be known. This is because it is necessary to transform the biomarker measure to *B* ~ Unif[0,1], such that Π(*B*) is the mean of *π*(*B*) in the range (*B*,1). We are primarily interested in this mean (or overall) response rate in the subpopulation, even though if a threshold of *T* is chosen, some subjects with biomarker quantile (*B*) values >*T* will have *π*(*B*) below Π(*T*). In general, these response rates are high enough that these subjects will still gain clinical benefit from the treatment, but this may not be the case if the logistic biomarker-response curve happens to be very steep, or if there are large gaps between the proposed threshold values. We strongly recommend studying both the *π*(*B*) and Π(*B*) curves carefully before choosing a threshold at the end of the study.

The choice of operating parameters in any particular scenario will depend on the circumstances and objective of that individual study, although we can offer some general guidelines, and many of these can be investigated further using the R [20] simulation code provided in the Supplementary material Section S2. The reference response rate should be based on clinical significance and a stage 1 threshold chosen to be close to the best prior estimate of *T*_H_, the smallest value of *B* at which the response rate would give a significant hypothesis test result. The size of the study should be set considering the power this would provide over a range of probable scenarios which can be estimated through simulation, and it is recommended to use equal stage 1 and stage 2 sample sizes (Section 3.4 and Supplementary material Section S4). It is important to take into account the potential number of participants that may have to be screened as a result of changing the threshold in the adaptive recruitment.

We proposed alternative implementations of the adaptive design, all with a main target power (1-*β*) and, upon failing to reach this target, each of which either stops at the interim or moves to the maximum threshold if it has predicted power ≥*γ*. The adaptive designs explored through simulation in Section 3 all used 1-*β* = 0.8, and AD2 and AD3 always had *γ* = 0.5 and *γ* = 0 respectively. Different values for 1-*β* and *γ* than those given here could be used, and the different costs of continuing to stage 2 in a null scenario, false positive and true negative outcomes need to be weighed against each other when deciding on the study design.

### 5.2 Observing powers well below the specified target power

The key components of our methodology are the specification of a target power, 1-*β*, and the choice of a stage 2 threshold based on achieving that power. However, in our simulations, we found that even when a target power of 1-*β* was predicted, we may in fact observe a much lower power. We attempt to explain this now.

Consider that at the interim, for each $t_{2,\text{k}}^{*},$ there is a true binomial probability of observing at least *X*_H,2_ = *X*_H_ − *X*_ob,1_ responses in stage 2. This probability is unknown, so it is estimated using the stage 1 data and the beta-binomial distribution. Consider that $t_{2,\text{k}}^{*}$ is the threshold which actually has binomial probability just >0.8, so it is the ‘ideal’ choice for *t*_2_. This probability could be underestimated or overestimated by the beta-binomial prediction. If it is overestimated, the study will definitely continue to stage 2, either using $t_{2,\text{k}}^{*}$ or a smaller value in $t_{2,}^{*}$ with predicted power also >0.8 (which must also be an overestimate). If it is underestimated, then a larger value in $t_{2}^{*}$ will be sought, which has predicted power >0.8. In some cases, no such value will exist, meaning, the study will be terminated. Therefore, more studies will reach completion in which the predicted power was an overestimate of the true binomial probability than an underestimate. For a fixed *t*_1_, this effect becomes more extreme when *T* is larger, as *X*_ob,1_ is likely to be smaller, and therefore *X*_H,2_ is larger. The only way to not include a stopping rule is to use a design like AD3, where the study continues at a threshold with a predicted power <1-*β*, and this also results in observed power <1-*β*.

### 5.3 Extensions to the CBATT design

There are several ways of extending the methodology presented here. One would be to use multiple interim analyses, which would improve the results if a poor choice for *t*_1_ had been made. If very little was known prior to the study to inform the parameter choices, for example, this could be implemented through an early interim with no stopping and then another half way through the remaining samples. This could be extended to more analyses still, even continuous updating, but further simulations would be required to evaluate how much value an approach such as this adds.

A way to decrease the number of studies stopped at the interim would be not to fix *S*_2_ in advance. In a study of this design, several potential sample sizes for stage 2, *S*_2_*, would have to be considered, and the powers for different combinations of (t_2_*, *S*_2_*) would be compared. Choosing the optimum combination of (*t*_2_*, *S*_2_*) would require a trade-off optimisation between multiple parameters, including power, study cost and duration and the accuracy of the final estimate of the threshold.

The design could be adapted to different types of outcome, such as continuous (e.g. tumour shrinkage or a pharmacodynamic measurement) or time-to-event (e.g. number of days from baseline until either disease progression occurs or follow-up is discontinued). The use of beta-binomial prediction is limited to binomial outcomes and is simplified through the application of the convenient conjugate-prior model. For a normally distributed outcome, conjugate-prior normal and gamma distributions could be used for the mean and variance parameters. For an outcome where a conjugate-prior model is not available, MCMC methods could be used to carry out the interim analysis and different appropriate models applied to these data types.

A control arm could be included in the study design with little adaptation of the method, enabling comparison in a subgroup between the novel treatment and a placebo or standard of care. Initial investigation of such a method has shown feasibility, but gains in power are limited due to the increased uncertainty from estimating the biomarker response curve in both the experimental arm and also the control arm.

Several other variations on the design could be adopted to address the exact aims of a specific trial. For example, including analysis of co-primary endpoints or treating stage 2 as an independent study.

### 5.4 Conclusions

We have presented a methodology for early-phase clinical trials which allows for flexibility when a predictive biomarker is hypothesised but *a priori* cannot be used to separate subjects into ‘positive’ and ‘negative’ subsets with confidence. This design includes a test for treatment efficacy in a subset of the population as well as estimation of the biomarker threshold defining ‘positive’ subjects. Hence, the continuous biomarker-adaptive threshold trial efficiently combines two objectives which may otherwise be investigated separately, increasing power over alternative methods.

## Supporting information

Additional supporting information can be found in the online version of this article at the publisher’s website.

Supplementary material

## Figures and Tables

**Table I:** Results from 5000 simulation iterations using adaptive design 1 and fixed design 1 with *S*_1_ = *S*_2_ = 50, *t*_1_ = 0.5, *ρ* = 0.4 and *α* = 0.05 in scenarios where *H*_0_ is true, but the maximum Π only just fails to reach *ρ*. Here, the proportion of significant simulations is a type-I error rate.

Π(0)	Proportion significant/type-I error rate (overall)		Proportion significant/type-I error rate (completed studies)^a^		Stopping rate		Ratio of subjects screened (AD1 to FD1)	
	
	Adaptive design 1	Fixed design 1	Adaptive design 1	Fixed design 1	Adaptive design 1	Fixed design 1	Overall	Completed studies
0.39	0.018	0.029	0.198	0.131	0.908	0.781	0.94	1.27
0.38	0.015	0.026	0.186	0.124	0.919	0.791	0.94	1.33
0.36	0.006	0.012	0.108	0.078	0.941	0.843	0.97	1.54
0.34	0.004	0.005	0.068	0.044	0.947	0.877	0.99	1.61
0.29	0.001	0.001	0.032	0.024	0.975	0.950	1.04	2.33
0.16	0	0	0	0	0.994	0.999	1.04	4.02

a Within the subset of studies not stopped at the interim, the proportion which resulted in a significant hypothesis test result.

**Table II:** Results from 5000 simulation iterations using adaptive design 1 and fixed design 1 with *S*_1_ = *S*_2_ = 50, *t*_1_ = 0.5, ρ = 0.4, α = 0.05 and *R*_H_ = *X*_H_ / *S* = 0.49 in scenarios with a biomarker effect and where *H*_A_ is true. Here, the proportion of significant simulations is power.

*δ*_1_	*T s.t.* Π (*T*)=*ρ*	*T*_H_ *s.t.* Π (*T*_H_)=*R*_H_	Π(0)	Π(0.95)	Proportion significant/power (overall)		Proportion significant/power (completed studies)^a^		Stopping rate		Ratio of subjects screened (AD1 to FD1)	
			
					Adaptive design 1	Fixed design 1	Adaptive design 1	Fixed design 1	Adaptive design 1	Fixed design 1	Overall	Completed studies
3	0.8	n/a	0.20	0.46	0.002	<0.001	0.067	0.024	0.967	0.984	1.16	3.21
6	0.8	0.93	0.13	0.51	0.002	0	0.069	0	0.974	>0.999	1.18	3.99
9	0.8	0.89	0.11	0.56	0.002	0	0.044	n/a^b^	0.964	1^b^	1.28	4.38^b^
3	0.6	0.85	0.24	0.53	0.036	0.008	0.273	0.069	0.866	0.890	1.36	2.42
6	0.6	0.74	0.19	0.65	0.148	0.001	0.503	0.020	0.706	0.941	2.24	2.83
9	0.6	0.71	0.17	0.74	0.350	0.001	0.717	0.014	0.512	0.958	3.14	2.83
3	0.5	0.76	0.27	0.56	0.110	0.037	0.448	0.153	0.755	0.760	1.49	2.22
6	0.5	0.65	0.22	0.71	0.384	0.033	0.729	0.144	0.474	0.771	2.28	2.21
9	0.5	0.62	0.21	0.81	0.669	0.039	0.831	0.161	0.195	0.755	2.80	2.04
3	0.4	0.66	0.29	0.60	0.228	0.145	0.570	0.340	0.600	0.573	1.41	1.76
6	0.4	0.56	0.26	0.76	0.623	0.237	0.795	0.447	0.217	0.469	1.85	1.67
9	0.4	0.53	0.24	0.86	0.827	0.336	0.866	0.562	0.046	0.402	1.74	1.43
3	0.23	0.5	0.34	0.66	0.477	0.510	0.734	0.699	0.350	0.271	1.20	1.33
6	0.33	0.5	0.28	0.79	0.732	0.522	0.824	0.704	0.111	0.259	1.45	1.35
9	0.35	0.5	0.26	0.89	0.844	0.599	0.856	0.766	0.014	0.218	1.33	1.20
3	0.2	0.48	0.35	0.66	0.515	0.586	0.750	0.758	0.314	0.227	1.17	1.28
6	0.2	0.38	0.33	0.84	0.830	0.913	0.847	0.968	0.020	0.057	1.03	1.01
9	0.2	0.36	0.32	0.93	0.859	0.983	0.860	0.996	0.001	0.013	0.88	0.88
3	0.02	0.3	0.397	0.72	0.872	0.599	0.880	0.766	0.112	0.218	1.04	1.20
6	0.10	0.3	0.36	0.87	0.895	0.990	0.899	0.997	0.005	0.007	0.85	0.85
9	0.13	0.3	0.35	0.95	0.901	0.997	0.901	>0.999	0	0.002	0.81	0.81

a Within the subset of studies not stopped at the interim, the proportion which resulted in a significant hypothesis test result.

b In this set of simulations, all studies with the fixed threshold design were stopped at the interim so there are no completed studies; however, the ratio of screened subjects is calculated based on any theoretical fixed design study that was not stopped having screened 200 subjects.

**Table III:** Results from 5000 simulation iterations using adaptive design 1 and fixed design 1 with *S*_1_ = *S*_2_ = 50, *t*_1_ = 0.5, *ρ* = 0.4 and *α* = 0.05 in scenarios with no biomarker effect and therefore a constant response rate.

Π	Proportion significant (overall)		Proportion significant (completed studies)^a^		Stopping rate		Ratio of subjects screened (AD1 to FD1)	
	
	Adaptive design 1	Fixed design 1	Adaptive design 1	Fixed design 1	Adaptive design 1	Fixed design 1	Overall	Completed studies
0.35	<0.001	0.001	0.016	0.018	0.975	0.934	0.99	1.56
0.40	0.022	0.033	0.210	0.135	0.897	0.758	0.93	1.23
0.42	0.049	0.078	0.343	0.226	0.857	0.668	0.89	1.16
0.50	0.409	0.569	0.843	0.760	0.515	0.251	0.81	0.94
0.55	0.729	0.872	0.971	0.938	0.249	0.071	0.79	0.84
0.65	0.982	0.998	1	>0.999	0.018	0.001	0.76	0.76

a Within the subset of studies not stopped at the interim, the proportion which resulted in a significant hypothesis test result.

**Table IV:** Results of hypothesis tests (*H*_A_: RR > 0.65) on subsets (*S* = 70) of subjects on hormonal therapy from the German Breast Study Group.

Recruitment	Responders/total sample size	Hypothesis test *p*-value	Threshold estimate (75% CI) fmol/mg
Adaptive threshold (CBATT with *t*_1_ = 0.35)	53 / 70	0.037	4 (0, 15)
Fixed threshold (*t* = 0.35)	48 / 70	0.312	8 (1, 20)
Full available data	96 / 176	n/a	11 (3, 32)

The subsets were ‘recruited’ with either the CBATT or fixed threshold design. Final threshold estimates and 75% confidence intervals from these subsets are also included, as well as for the full 176 subjects in the dataset who were on hormonal therapy and whose survival data were not censored before 1500 days.
