# Supplementary material for: An adaptive design for updating the threshold value of a continuous biomarker
Source: Stat Med. Author manuscript; Available in PMC 2017 Apr 3. (PMC5378309; doi:10.1002/sim.7042)
Supplement: Supplementary material [file NIHMS71921-supplement-Supporting_Information.docx]

**An Adaptive Design for Updating the Threshold Value of a Continuous Biomarker - Supplementary Material**

**S1: Derivation of Π(B) function**

Π(*B*), is the average value of π(*B*) in the range of biomarker quantiles (*B*, 1), and this can be found using calculus:

$$\Pi\left( B \right)= \frac{1}{1-B}\int_{B}^{1} \pi\left( B \right)dB$$

$$= \frac{1}{1-B}\int_{B}^{1} \frac{exp(\delta_{0}+\delta_{1}B)}{1+exp(\delta_{0}+\delta_{1}B)}dB.$$

To aid this calculation, we can use $\frac{d}{dB}\exp\left( \delta_{0}+\delta_{1}B \right)=\delta_{1}exp(\delta_{0}+\delta_{1}B)$:

$$\Pi\left( B \right)= \frac{1}{\delta_{1}(1-B)}\int_{B}^{1} \frac{1}{1+exp(\delta_{0}+\delta_{1}B)}dexp(\delta_{0}+\delta_{1}B)$$

$$= \frac{1}{\delta_{1}(1-B)}\left[ ln(1+exp(\delta_{0}+\delta_{1}B)) \right]_{B=B}^{B=1}$$

$$=\ln\left( \frac{1+exp(\delta_{0}+\delta_{1})}{1+exp(\delta_{0}+\delta_{1}B)} \right)/\delta_{1}\left( 1-B \right).$$


**Supplementary Figure 1: Histograms of the 1000 estimates of Π(*B*) using** $\left( {\tilde{\boldsymbol{\delta}}}_{\boldsymbol{0}}\boldsymbol{,}{\tilde{\boldsymbol{\delta}}}_{\boldsymbol{1}} \right)$**from the MVN distribution at various values of *B*. The fitted beta distributions are also plotted, along with the MCMC densities. The data comes from a single simulation run with *S*_1_ = 50, *t*_1_ = 0.5, *T =* 0.8, *δ*_1_ = 6, *R*= 0.4.**

**S2: R simulation code**

########################################

### Simulation code for distribution ###

### Amy Spencer 20/1/16 ###

### Developed in R version 3.0.2 ###

########################################

### This function simulates stage 1 of a study and then predicts the ###

### probability of achieving a significant hypothesis test in stage 2 if ###

### any of the values in t2.star were to be used as a stage 2 threshold. ###

### It then simulates stage 2 for each of those t2 values. This then ###

### allows comparison between using different rules for the choice of the ###

### stage 2 threshold using the summary function below. ###

# Specify the following parameters:

# n the number of simulations

# ss1 sample size in stage 1

# ss2 sample size in stage 2

# t1 the fixed threshold for stage 1

# t2.star vector of values from which the threshold in stage 2 will be

# chosen

# targetRR rho, the target response rate which the hypothesis test will use

# alpha significance level

# Thr true threshold (either this or delta0 must be specified)

# delta0 intercept of the true logisitic curve (either this or Thr must be

# specified; if Thr=0, this must be specified as well)

# delta1 slope of the true logisitic curve (must be specified)

two.stage.grid<-function(n=5000,ss1=50,ss2=50,t1=0.7,t2.star=seq(0,0.95,0.05),

targetRR=0.4,alpha=0.05,Thr=0.5,delta0=NA,delta1=7){

### load necessary packages ###

library("MASS")

library("VGAM")

library("fitdistrplus")

### find target number of responses for total sample size ###

target<-qbinom(alpha,ss1+ss2,targetRR,lower.tail=F)+1

### If Thr is specified rather than delta0, find delta0 ###

if(is.na(delta0)==TRUE){

f<-function(x){

log((1+exp(x+delta1))/(1+exp(x+delta1*Thr)))/(delta1*(1-Thr))-targetRR

}

delta0<-uniroot(f,interval=c(-50,50))$root

}

### set up matrices and vectors for outputs of simulations ###

coef.est<-matrix(NA,n,2)

coef.vcov<-matrix(NA,n,3)

s1pval<-rep(NA,n)

power<-matrix(NA,n,length(t2.star))

significant<-matrix(0,n,length(t2.star))

responses1<-rep(NA,n)

responses2<-matrix(NA,n,length(t2.star))

T.hat<-matrix(NA,n,length(t2.star))

CI95L<-matrix(NA,n,length(t2.star))

CI95U<-matrix(NA,n,length(t2.star))

CI90L<-matrix(NA,n,length(t2.star))

CI90U<-matrix(NA,n,length(t2.star))

CI75L<-matrix(NA,n,length(t2.star))

CI75U<-matrix(NA,n,length(t2.star))

CI50L<-matrix(NA,n,length(t2.star))

CI50U<-matrix(NA,n,length(t2.star))

### Simulation loop ###

for(j in 1:n){

### simulate ss1 subjects above BM threshold = t1 for each study ###

BMs1<-runif(ss1,t1,1)

trt.logit<-delta0+delta1*BMs1

trt.prob<-exp(trt.logit)/(1+exp(trt.logit))

trials1<-rbinom(ss1,size=1,prob=trt.prob)

responses1[j]<-sum(trials1)

### logistic regression ###

BM<-BMs1

trial<-trials1

model1a<-glm(trial ~ BM, family = binomial)

s1pval[j]<-summary(model1a)$coefficients[2,4]

### find response rate *above* each possible threshold (group.prob) ###

new.BM<-t2.star

int<-model1a[[1]][1]

slp<-model1a[[1]][2]

group.prob<-log((1+exp(int+slp))/(1+exp(int+slp*new.BM)))/(slp*(1-new.BM))

### simulate data using fitted parameter values from regresssion ###

mu<-model1a$coefficients

Sigma<-vcov(model1a)

sim.curves<-mvrnorm(n = 1000, mu, Sigma, tol = 1e-6, empirical = TRUE,

EISPACK = FALSE)

coef.est[j,]<-mu

coef.vcov[j,]<-c(Sigma[1,],Sigma[2,2])

### find above theshold response rate for simulated data ###

sim.fitted<-matrix(NA,length(t2.star),1000)

for(i in 1:1000){

int<-sim.curves[i,1]

slp<-sim.curves[i,2]

sim.fitted[,i]<-log((1+exp(int+slp))/(1+exp(int+slp*new.BM)))/

(slp*(1-new.BM))

}

### find beta-binomial dists and run a stage 2 using each threshold ###

if(sum(sim.fitted=="NaN")==0){

for(i in 1:length(t2.star)){

fitted<-fitdist(sim.fitted[i,which(sim.fitted[i,]<=1)],"beta",

method = "mme",start=list(shape1=1,shape2=1))

power[j,i]<-1-pbetabinom.ab(target-responses1[j]-1, ss2,

fitted[[1]][1], fitted[[1]][2])

### simulate ss2 subjects above BM threshold = t2 for each study ###

BMs2<-runif(ss2,t2.star[i],1)

trt.logit<-delta0+delta1*BMs2

trt.prob<-exp(trt.logit)/(1+exp(trt.logit))

trials2<-rbinom(ss2,size=1,prob=trt.prob)

BM<-c(BMs1,BMs2)

trial<-c(trials1,trials2)

responses<-sum(trial)

responses2[j,i]<-responses-responses1[j]

if(responses>=target){

significant[j,i]<-1

}

### analyse stage 1 and stage 2 data together ###

model1b<-glm(trial ~ BM, family = binomial)

new.BM<-seq(0.00,0.99,0.01)

int<-model1b[[1]][1]

slp<-model1b[[1]][2]

new.logit2<-int+slp*new.BM

new.prob2<-exp(new.logit2)/(1+exp(new.logit2))

group.prob2<-log((1+exp(int+slp))/(1+exp(int+slp*new.BM)))/

(slp*(1-new.BM))

if(max(group.prob2)>=targetRR){

T.hat[j,i]<-new.BM[min(which(group.prob2>=targetRR))]

} else { T.hat[j,i]=1 }

### simulate data using fitted parameter values from regresssion ###

mu2<-model1b$coefficients

Sigma2<-vcov(model1b)

sim.curves2<-mvrnorm(n = 1000, mu2, Sigma2, tol = 1e-6,

empirical = TRUE, EISPACK = FALSE)

### find above theshold response rate for simulated data ###

sim.fitted2<-matrix(NA,100,1000)

for(k in 1:1000){

int<-sim.curves2[k,1]

slp<-sim.curves2[k,2]

sim.fitted2[,k]<-log((1+exp(int+slp))/(1+exp(int+slp*new.BM)))/

(slp*(1-new.BM))

}

T.RR<-rep(NA,1000)

for(k in 1:1000){

if(max(sim.fitted2[,k])>=targetRR){

T.RR[k]<-new.BM[min(which(sim.fitted2[,k]>=targetRR))]

} else { T.RR[k]=1 }

}

### credible intervals for estimated thresold ###

CI95L[j,i]<-quantile(T.RR,0.025)

CI95U[j,i]<-quantile(T.RR,0.975)

CI90L[j,i]<-quantile(T.RR,0.05)

CI90U[j,i]<-quantile(T.RR,0.95)

CI75L[j,i]<-quantile(T.RR,0.125)

CI75U[j,i]<-quantile(T.RR,0.875)

CI50L[j,i]<-quantile(T.RR,0.25)

CI50U[j,i]<-quantile(T.RR,0.75)

} # End for t2 loop

} # End if loop

### if study significant after stage 1(could be stopped for efficacy) ###

if(responses1[j]>=target){

power[j,]=1

significant[j,]=1

}

} #End simulation loop

full.results<-list(n=n,ss1=ss1,ss2=ss2,t1=t1,t2.star=t2.star,

targetRR=targetRR,alpha=alpha,Thr=Thr,delta0=delta0,delta1=delta1,

coef.est=coef.est,coef.vcov=coef.vcov,s1pval=s1pval,power=power,

significant=significant,responses1=responses1,responses2=responses2,

T.hat=T.hat,CI95L=CI95L,CI95U=CI95U,CI90L=CI90L,CI90U=CI90U,CI75L=CI75L,

CI75U=CI75U,CI50L=CI50L,CI50U=CI50U)

return(full.results)

} #End function

# Outputs:

# n see input parameters

# ss1 see input parameters

# ss2 see input parameters

# t1 see input parameters

# t2.star see input parameters

# targetRR see input parameters

# alpha see input parameters

# Thr see input parameters

# delta0 see input parameters; if this was not specified it will have

# been calculated using Thr and delta1

# delta1 see input parameters

# coef.est a n*2 matrix of the estimated intercept (column 1) and slope

# (column 2) of the logistic model using the simulated data

# from stage 1 (n simulations)

# coef.vcov a n*3 matrix of the estimated intercept and slope variances

# (columns 1 and 3) and covariance (column 2) of the logistic

# model using the simulated data from stage 1 (n simulations)

# s1pval a vector (of length n) of the p-values of the biomarker

# slope terms in the logistic model after stage 1

# power a n*length(t2.star) matrix of predicted powers in the n

# simulations using the different potential stage 2 thresholds

# in t2.star

# significant a n*length(t2.star) matrix of 1s and 0s, indicating whether

# the n-th simulation was significant, when the different

# values in t2.star were used as a stage 2 threshold

# responses1 a vector (of length n) indicating the number of responders

# in stage 1 of the study

# responses2 a n*length(t2.star) matrix indicating the number of

# responders in stage 2 of the study, when the different

# values in t2.star were used as a stage 2 threshold

# T.hat a n*length(t2.star) matrix indicating the estimated

# threshold at the end of the study, when the different values

# in t2.star were used as a stage 2 threshold

# CI95L a n*length(t2.star) matrix of lower bounds for the 95%

# credible interval for the estimated threshold at the end of

# the study in the n simulations and using the different

# values in t2.star as a stage 2 threshold

# CI95U a n*length(t2.star) matrix of upper bounds for the 95%

# credible interval for the estimated threshold at the end of

# the study in the n simulations and using the different

# values in t2.star as a stage 2 threshold

# CI90L lower bounds for the 90% credible interval for the estimated

# threshold at the end of the study

# CI90U upper bounds for the 90% credible interval for the estimated

# threshold at the end of the study

# CI75L lower bounds for the 75% credible interval for the estimated

# threshold at the end of the study

# CI75U upper bounds for the 75% credible interval for the estimated

# threshold at the end of the study

# CI50L lower bounds for the 50% credible interval for the estimated

# threshold at the end of the study

# CI50U upper bounds for the 50% credible interval for the estimated

# threshold at the end of the study

###########################################################################

########################

### Summary function ###

########################

### This function summarises the output of the two.stage.grid function ###

### using the data that would have been obtained if a particular rule was ###

### used for choosing the stage 2 threshold. For a simple fixed threshold ###

### design without a stopping rule, specify s2thr=TSGobject$t1. For a ###

### fixed threshold design with a beta-binomial stopping rule, specify ###

### this and also specify FixBB. For a basic CBATT design, specify ###

### targetPow. If you also want to include the option to move to the ###

### maximum value of t2.star if all predicted powers < targetPow, specify ###

### minPow, the minimum value the predicted power for max(t2.star) should ###

### take to prevent the stopping (specifying 0 prevents stopping ###

### altogether). ###

# Specify the following parameters:

# TSGobject output of two.stage.grid simulation call

# FixBB for fixed threshold designs with a stopping rule based on the

# beta-binomial prediction, what value (in the range 0,1) should be

# used for the stopping rule?

# s2thr what value should be used for the stage 2 recruitment threshold?

# Usually you will want to use TSGobject$t1 for fixed design or NA

# for adaptive design

# targetPow 1-beta value for the adaptive design (used to choose stage 2

# threhsold)

# minPow optional additional adaptive design rule: should take a value

# between 0 and targetPow. If the predicted power is below

# targetPow for all thresholds, the stage 2 threshold will take the

# max(t2.star) value, but only if the predicted power at that

# threshold is >=minPow (otherwise stopped at interim)

# useS1 for trials with a stopping rule: if TRUE, summary statistics will

# be calculated using results from stage 1 of any study that is

# stopped early; if FALSE studies that are stopped early will be

# disregarded in the calculation of summary statistics

compare.sum<-function(TSGobject,FixBB=NA,s2thr=NA,targetPow=NA,minPow=NA,

useS1=FALSE){

pp<-TSGobject$power

op<-TSGobject$significant

op[op==0]<-2

stage1<-TSGobject$responses1

S1model<-TSGobject$coef.est

S1var<-TSGobject$coef.vcov

fullT.hat<-TSGobject$T.hat

fullT.hat[is.na(fullT.hat)]=1

fullBias<-fullT.hat-TSGobject$Thr

# Bias is difference between estimated and true threshold

fullCI50range<-TSGobject$CI50U-TSGobject$CI50L

# Length of 50% credible interval

result.type<-rep(NA,TSGobject$n)

T.hat<-rep(NA,TSGobject$n)

bias<-rep(NA,TSGobject$n)

CI50range<-rep(NA,TSGobject$n)

S2sample<-rep(NA,TSGobject$n)

### Option 1: Fixed design ###

if(is.na(s2thr)==F){

index<-which(round(TSGobject$t2.star,2)==s2thr)

# find the index of the threshold used in stage 2

S2sample<-rep(TSGobject$ss2/(1-s2thr),TSGobject$n)

# average subjects needed to be screened for stage 2

### 1a: No stopping rule ###

if(is.na(FixBB)){

result.type<-op[,index]

T.hat<-fullT.hat[,index]

bias<-fullBias[,index]

CI50range<-fullCI50range[,index]

}

### 1b: Beta-binomial stopping rule ###

if(is.na(FixBB)==F){

library("VGAM")

s1NonSig<-TSGobject$ss1-stage1 # subjects which didn't respond in s1

reqS2<-qbinom(0.05,TSGobject$ss1+TSGobject$ss2,TSGobject$targetRR,

lower.tail=F)+1-stage1

# number need to respond in stage 2 to get a significant result

for(i in 1:TSGobject$n){

shape1<-max(stage1[i],0.7)

shape2<-max(s1NonSig[i],0.001)

fixPow<-1-pbetabinom.ab(reqS2[i]-1, TSGobject$ss2, shape1, shape2)

### study stopped at interim if predicted power < chosen value ###

if(fixPow<FixBB){

result.type[i]<-3

S2sample[i]<-0 #average subjects needed to be screened in stage 2

if(useS1==TRUE){

library("MASS")

### calculate values using stage 1 data in stopped trials ###

new.BM<-seq(0.00,0.99,0.01)

int<-S1model[i,1]

slp<-S1model[i,2]

group.prob<-log((1+exp(int+slp))/(1+exp(int+slp*new.BM)))/

(slp*(1-new.BM))

if(max(group.prob)>=TSGobject$targetRR){

T.est<-new.BM[min(which(group.prob>=TSGobject$targetRR))]

} else { T.est=1 }

T.hat[i]<-T.est

bias[i]<-T.hat[i]-TSGobject$Thr

mu<-S1model[i,]

Sigma<-matrix(c(S1var[i,1:2],S1var[i,2:3]),2,2)

sim.curves<-mvrnorm(n = 1000, mu, Sigma, tol = 1e-6,

empirical = TRUE, EISPACK = FALSE)

sim.fitted<-matrix(NA,100,1000)

for(k in 1:1000){

int<-sim.curves[k,1]

slp<-sim.curves[k,2]

sim.fitted[,k]<-log((1+exp(int+slp))/(1+exp(int+slp*new.BM)))/

(slp*(1-new.BM))

}

T.RR<-rep(NA,1000)

for(k in 1:1000){

if(max(sim.fitted[,k])>=TSGobject$targetRR){

T.RR[k]<-new.BM[min(which(sim.fitted[,k] >=

TSGobject$targetRR))]

} else { T.RR[k]=1 }

}

CI50L<-quantile(T.RR,0.25)

CI50U<-quantile(T.RR,0.75)

CI50range[i]<-CI50U-CI50L

} # End if loop (useS1)

} # End if loop (stopped at interim)

### study continues to stage 2 if predicted power >= chosen value ###

if(fixPow>=FixBB){

result.type[i]<-op[i,index]

T.hat[i]<-fullT.hat[i,index]

bias[i]<-fullBias[i,index]

CI50range[i]<-fullCI50range[i,index]

} #End if loop (continues to stage 2)

} # End for loop (each simulation)

} # End if loop (stopping rule)

} # End if loop (fixed design)

### Option 2: Adaptive design ###

if(is.na(s2thr)){

for(i in 1:TSGobject$n){

if(max(pp[i,])>=targetPow){

index<-min(which(pp[i,]>=targetPow))

} else { index=NA }

# find the index of the threshold used in stage 2

### 2a: if target power is not achieved use max threshold ONLY IF ###

### above minimum power ###

if(is.na(pp[i,length(TSGobject$t2.star)])==F){

if(is.na(minPow)==F & is.na(index) &

pp[i,length(TSGobject$t2.star)]>=minPow){

index<-which(TSGobject$t2.star==max(TSGobject$t2.star))

}

}

if(is.na(index)){ # This indicates study is stopped (doesn't meet rules)

result.type[i]<-3

S2sample[i]<-0 #average subjects needed to be screened in stage 2

if(useS1==TRUE){

library("MASS")

### calculate values using stage 1 data in stopped trials ###

new.BM<-seq(0.00,0.99,0.01)

int<-S1model[i,1]

slp<-S1model[i,2]

group.prob<-log((1+exp(int+slp))/(1+exp(int+slp*new.BM)))/

(slp*(1-new.BM))

if(max(group.prob)>=TSGobject$targetRR){

T.est<-new.BM[min(which(group.prob>=TSGobject$targetRR))]

} else { T.est=1 }

T.hat[i]<-T.est

bias[i]<-T.hat[i]-TSGobject$Thr

mu<-S1model[i,]

Sigma<-matrix(c(S1var[i,1:2],S1var[i,2:3]),2,2)

sim.curves<-mvrnorm(n = 1000, mu, Sigma, tol = 1e-6,

empirical = TRUE, EISPACK = FALSE)

sim.fitted<-matrix(NA,100,1000)

for(k in 1:1000){

int<-sim.curves[k,1]

slp<-sim.curves[k,2]

sim.fitted[,k]<-log((1+exp(int+slp))/(1+exp(int+slp*new.BM)))/

(slp*(1-new.BM))

}

T.RR<-rep(NA,1000)

for(k in 1:1000){

if(max(sim.fitted[,k])>=TSGobject$targetRR){

T.RR[k]<-new.BM[min(which(sim.fitted[,k]>=TSGobject$targetRR))]

} else { T.RR[k]=1 }

}

CI50L<-quantile(T.RR,0.25)

CI50U<-quantile(T.RR,0.75)

CI50range[i]<-CI50U-CI50L

} # End if loop (useS1)

} # End if loop (stopping rule)

if(is.na(index)==FALSE){ # Study is not stopped

result.type[i]<-op[i,index]

T.hat[i]<-fullT.hat[i,index]

bias[i]<-fullBias[i,index]

CI50range[i]<-fullCI50range[i,index]

}

S2sample[i]<-TSGobject$ss2/(1-(index*0.05-0.05))

#average subjects needed to be screened in stage 2

} # End for loop (each simulation)

} # End if loop (adaptive design)

### Proportion of trials... ###

signif<-mean(result.type==1)

non.signif<-mean(result.type==2)

stopped<-mean(result.type==3)

### Estimation of T (based on both significant and non-significant)... ###

meanBias<-mean(bias,na.rm=TRUE)

sdT.hat<-sd(T.hat,na.rm=TRUE)

medBias<-median(bias,na.rm=TRUE)

iqrT.hat<-IQR(T.hat,na.rm=TRUE)

medCI50range<-median(CI50range,na.rm=TRUE)

S2sample[is.na(S2sample)]<-0

# for studies that were stopped, no subjects screened for stage 2

meanS2sample<-mean(S2sample)

output<-c(n=TSGobject$n,ss1=TSGobject$ss1,ss2=TSGobject$ss2,t1=TSGobject$t1,

t2.star=TSGobject$t2.star,targetRR=TSGobject$targetRR,

alpha=TSGobject$alpha,Thr=TSGobject$Thr,delta0=TSGobject$delta0,

delta1=TSGobject$delta1,signif=signif,non.signif=non.signif,

stopped=stopped,meanS2sample=meanS2sample,meanBias=meanBias,

sdT.hat=sdT.hat,medBias=medBias,iqrT.hat=iqrT.hat,

medCI50range=medCI50range)

return(output)

} # End function

# Outputs:

# n see input parameters for two.stage.grid

# ss1 see input parameters for two.stage.grid

# ss2 see input parameters for two.stage.grid

# t1 see input parameters for two.stage.grid

# t2.star see input parameters for two.stage.grid

# targetRR see input parameters for two.stage.grid

# alpha see input parameters for two.stage.grid

# Thr see input parameters for two.stage.grid

# delta0 see input parameters for two.stage.grid; if this was not

# specified it will have been calculated using Thr and delta1

# delta1 see input parameters for two.stage.grid

# signif proportion of simulations that had significant hypothesis

# test results

# non.signif proportion of simulations that had non-significant

# hypothesis test results

# stopped proportion of simulations that were stopped early at the

# interim

# meanS2sample mean number of subjects that need to be screened in order to

# recruit ss2 subjects with biomarker values above the stage 2

# threshold (or none if stopped early) in stage 2

# meanBias mean amount of bias in the final estimated threshold (over

# all simulations)

# sdT.hat standard deviation in the final estimated threshold (over

# all simulations)

# medBias median amount of bias in the final estimated threshold (over

# all simulations)

# iqrT.hat interquartile range in the final estimated threshold (over

# all simulations)

# medCI50range median length of the 50% credible interval for the estimated

# threshold at the end of the study (over all simulations)

############################################################################

#######################

### Small example 1 ###

#######################

# Non-null example: see table 2 in paper

testTSG<-two.stage.grid(n=50,ss1=50,ss2=50,t1=0.5,t2.star=seq(0,0.95,0.05),

targetRR=0.4,alpha=0.05,Thr=0.4,delta0=NA,delta1=6)

#Fixed designs

compare.sum(TSGobject=testTSG,FixBB=NA,s2thr=testTSG$t1,targetPow=NA,

minPow=NA,useS1=FALSE) #FD2

compare.sum(TSGobject=testTSG,FixBB=0.2,s2thr=testTSG$t1,targetPow=NA,

minPow=NA,useS1=FALSE) #FD1

compare.sum(TSGobject=testTSG,FixBB=0.2,s2thr=testTSG$t1,targetPow=NA,

minPow=NA,useS1=TRUE) #FD1

#Adaptive designs

compare.sum(TSGobject=testTSG,FixBB=NA,s2thr=NA,targetPow=0.8,

minPow=NA,useS1=FALSE) #AD1

compare.sum(TSGobject=testTSG,FixBB=NA,s2thr=NA,targetPow=0.8,

minPow=NA,useS1=TRUE) #AD1

compare.sum(TSGobject=testTSG,FixBB=NA,s2thr=NA,targetPow=0.8,

minPow=0.5,useS1=FALSE) #AD2

#######################

### Small example 2 ###

#######################

# Null example: see table 1 in paper

testTSG2<-two.stage.grid(n=50,ss1=50,ss2=50,t1=0.5,t2.star=seq(0,0.95,0.05),

targetRR=0.4,alpha=0.05,Thr=0,delta0=-0.71,delta1=0.3)

#Fixed designs

compare.sum(TSGobject=testTSG2,FixBB=NA,s2thr=testTSG$t1,targetPow=NA,

minPow=NA,useS1=FALSE) #FD2

compare.sum(TSGobject=testTSG2,FixBB=0.2,s2thr=testTSG$t1,targetPow=NA,

minPow=NA,useS1=FALSE) #FD1

compare.sum(TSGobject=testTSG2,FixBB=0.2,s2thr=testTSG$t1,targetPow=NA,

minPow=NA,useS1=TRUE) #FD1

#Adaptive designs

compare.sum(TSGobject=testTSG2,FixBB=NA,s2thr=NA,targetPow=0.8,

minPow=NA,useS1=FALSE) #AD1

compare.sum(TSGobject=testTSG2,FixBB=NA,s2thr=NA,targetPow=0.8,

minPow=NA,useS1=TRUE) #AD1

compare.sum(TSGobject=testTSG2,FixBB=NA,s2thr=NA,targetPow=0.8,

minPow=0.5,useS1=FALSE) #AD2

**Supplementary Figure 2: The π(*B*) logistic curves (dashed lines) and Π(*B*) subset response rate curves (solid lines) for the simulated scenarios given in Table 2. The pale dotted lines indicate *T* and *T_H_*, the thresholds of *B* above which response rates of *ρ* = 0.4 and *R_H_* = 0.49 occur.**

**S3: Exact power and stopping rate for scenarios where Π is constant**

Suppose that there is no stopping, for example, AD3 or FD2. In this case P(*X_ob_* ≥ *X_H_*) can be obtained from the binomial distribution $X_{ob}\sim\mathrm{Bi}(S,\Pi)$, because this will be the true binomial distribution no matter what recruitment thresholds are used.

**Table S1: Comparing the true binomial probability of a significant hypothesis test result to estimates from 5000 simulations using adaptive design 3 and fixed design 2.**

| π | Proportion significant | | |
| --- | --- | --- | --- |
|  | Binomial probability | Adaptive design 3 (5000 simulations) | Fixed design 2 (5000 simulations) |
| 0.35 | 0.003 | 0.001 | 0.001 |
| 0.40 | 0.042 | 0.043 | 0.038 |
| 0.42 | 0.094 | 0.094 | 0.090 |
| 0.50 | 0.618 | 0.614 | 0.624 |
| 0.55 | 0.904 | 0.916 | 0.906 |
| 0.65 | >0.999 | 1 | >0.999 |

Now consider adaptive design 1, so stopping will occur if no values in $\boldsymbol{t}_{\boldsymbol{2}}^{\boldsymbol{*}}$ have a predicted power ≥ $1-\beta$. To do this we must assume that the fitted model at the interim has $\hat{\Pi}$ constant, although not necessarily $\hat{\Pi}=\Pi$. This assumption is met, on average, as $\hat{\delta}_{1}\sim N(\delta_{1}=0,V)$ (asymptotically). First, calculate the probability of observing each potential value $X_{ob,1}\in(0,S_{1})$ in stage 1 using $X_{ob,1}\sim\mathrm{Bi}(S_{1},\Pi)$. As $\hat{\Pi}$ is constant, each $t_{2,k}^{*}$ will have the same beta-binomial prediction distribution, $X_{t_{2,k}^{*}}\sim\mathrm{BBi}(S_{2},X_{ob,1},S_{1}-X_{ob,1})$, and for each$X_{ob,1}$, you can calculate ${1-\beta'}_{t_{2,k}^{*}}={P(X}_{t_{2,k}^{*}}\geq{(X}_{H}-X_{ob,1}))$. Stopping will occur if this probability is less than $1-\beta$, so the total stopping rate is

$$P_{\mathrm{STOP}}=\sum_{X_{ob,1}=0}^{S_{1}} P{(X}_{ob,1})*{P(X}_{t_{2,k}^{*}}<{(X}_{H}-X_{ob,1})).$$

**Table S2: Comparing the true underlying probability (*P*_SIG_) of a significant hypothesis test result for adaptive design 1 and stopping rate (*P*_STOP_) to estimates from 5000 simulations.**

| π | Proportion significant (overall, adaptive design 1) | | Stopping rate (adaptive design 1) | |
| --- | --- | --- | --- | --- |
|  | $P_{\mathrm{SIG}}$ | 5000 Simulations | $P_{\mathrm{STOP}}$ | 5000 Simulations |
| 0.35 | 0.002 | <0.001 | 0.945 | 0.975 |
| 0.40 | 0.026 | 0.022 | 0.841 | 0.897 |
| 0.42 | 0.059 | 0.049 | 0.777 | 0.857 |
| 0.50 | 0.445 | 0.409 | 0.432 | 0.515 |
| 0.55 | 0.738 | 0.729 | 0.226 | 0.249 |
| 0.65 | 0.974 | 0.982 | 0.026 | 0.018 |

Next, for each $X_{ob,1}$, we calculate the probability of a significant hypothesis test result, conditional on a trial reaching stage 2 (not being stopped at the interim). Where ${X_{H,2}=X}_{H}-X_{ob,1}$, this is ${P(X}_{ob,2}\geq X_{H,2}| {{X_{ob,2}\sim\mathrm{Bi}\left( S_{2},\Pi\right),\beta}^{'}}_{t_{2,k}^{*}}\geq\beta)$. So the total probability of a significant test result is

$$P_{\mathrm{SIG}}=\sum_{X_{ob,1}=0}^{S_{1}} P{(X}_{ob,1})*{P(X}_{t_{2,k}^{*}}\geq X_{H,2})*{P(X}_{ob,2}\geq X_{H,2}).$$

**S4: Changing the timing of the interim analysis**

To investigate whether it could be advantageous to use *S*_2_ ≠ *S*_1_, we carried out simulations changing the timing of the interim analysis but leaving all other parameters the same. Supplementary Figure 3(a) contains results for several sets of simulations where the true underlying model had *T* = 0.6 and *δ*_1_ = 6, the stage 1 threshold was set at *t*_1_ = 0.7, and the total sample size was 80, 200 or 320. We see that as *S* increases, the power increases, but that the maximum power for each *S* is always achieved when *S*_2_ ≈ *S*_1_. This achieves a balance between having enough information at the interim to accurately determine *t*_2_ and having a large enough stage 2 to allow any change of threshold to have an impact. Further simulations showed us that this trend generally occurs when *t*_1_ is close to *T_H_*, as is the case here (*T_H_* is between 0.68 and 0.76), but differs with the relationship between *t*_1_ and *T_H_*.

**(a)** **
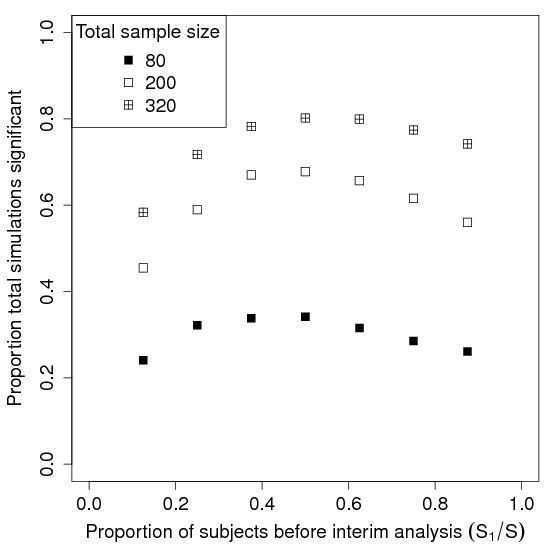
(b)
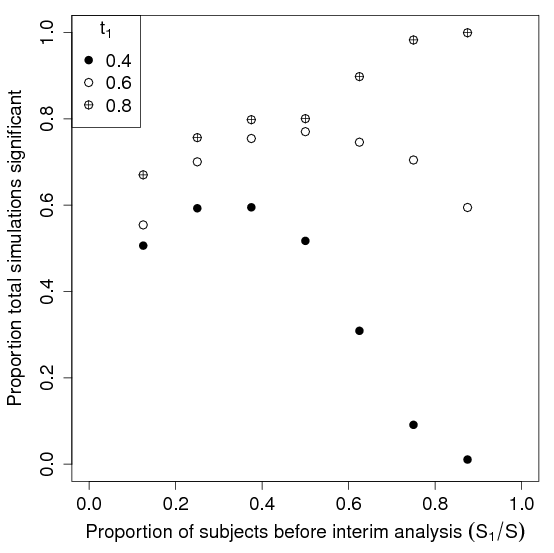
**

**Supplementary Figure 3: The proportion of 5000 iterations of a simulated study (adaptive design 1) that produce significant hypothesis test results dependent on when the interim analysis is carried out. We also consider how this varies with (a) total sample size (*S*), when the data is generated from a logistic model with *T* = 0.6 and *δ*_1_ = 6, and *t*_1_ = 0.7 is used; and (b) stage 1 threshold (*t*_1_), when the data is generated from a logistic model with *T* = 0.5 and *δ*_1_ = 6, and *S* = 200 is used. Other parameters are fixed at *α* = 0.05, 1-*β* = 0.8, *R* =0.4.**

The difference in the relationship between *S*_1_ and power for some values of *t*_1_, where the true model has *δ*_1_ = 6 and *T* = 0.5, is demonstrated in Supplementary Figure 3(b), this time using *S* = 200 so that *T_H_* = 0.61. We see that when *t*_1_ = 0.6 the change in the proportion of significant studies with the timing of the interim analysis is similar to that in the examples shown in Supplementary Figure 3(a), because this is another case where *t*_1_ is close to *T_H_*. However, the trends are quite different for the other values of *t*_1_ and can be explained thus. When *t*_1_ = 0.4, the power increases with *S*_1,_ due to the increase in information about the underlying model being able to give a better idea of what value should be used for *t*_2_. This is only the case up to maximum at around *S*_1_ = 75 (*S*_1_/*S* = 0.375), after which there occurs a rapid decrease in power, because with such a low *t*_1_, *X_ob,_*_1_ will be low and *S*_2_ is now too small to regularly produce *X_ob,_*_2_ such that *X_ob,_*_1_ + *X_ob,_*_2_ = *X_ob_* > *X_H_*, even using a large *t*_2_. On the other hand, when *t*_1_ = 0.8, the increase in power with *S*_1_ is continuous, but has an unusual trajectory. There is a plateau at a power of approximately 1-*β* = 0.8 around *S*_1_/*S* = 0.5, after which the power begins to increase again. At the plateau, it appears that most simulations have chosen a *t*_2_ at which the predicted power is suitable. With higher values of *S*_1_ and also *t*_1_ >> *T*, *X_ob,_*_1_ will already be so large that even small values of *t*_2_ will produce enough responses so that *X_H_* is exceeded with probability > 1-*β*.
